# Bayesian poisson regression tensor train decomposition model for learning mortality pattern changes during COVID-19 pandemic

**DOI:** 10.1080/02664763.2024.2411608

**Published:** 2024-10-10

**Authors:** Wei Zhang, Antonietta Mira, Ernst C. Wit

**Affiliations:** a Faculty of Informatics, Università della Svizzera italiana, Lugano, Switzerland; b Faculty of Economics, Euler Institute, Università della Svizzera italiana, Lugano, Switzerland; c Department of Science and High Technology, Insubria University, Como, Italy

**Keywords:** Bayesian inference, COVID-19, mortality, tensor decomposition

## Abstract

COVID-19 has led to excess deaths around the world. However, the impact on mortality rates from other causes of death during this time remains unclear. To understand the broader impact of COVID-19 on other causes of death, we analyze Italian official data covering monthly mortality counts from January 2015 to December 2020. To handle the high-dimensional nature of the data, we developed a model that combines Poisson regression with tensor train decomposition to explore the lower-dimensional residual structure of the data. Our Bayesian approach incorporates prior information on model parameters and utilizes an efficient Metropolis-Hastings within Gibbs algorithm for posterior inference. Simulation studies were conducted to validate our approach. Our method not only identifies differential effects of interventions on cause-specific mortality rates through Poisson regression but also provides insights into the relationship between COVID-19 and other causes of death. Additionally, it uncovers latent classes related to demographic characteristics, temporal patterns, and causes of death.

## 1. Introduction

Following the outbreak, COVID-19 has had profound consequences on various aspects such as economics, environment, and politics [1,4,15]. Focusing on its impacts on health and health systems, excess mortality due to the pandemic is under scrutiny as it provides an overall picture of the pandemic's impact on human health, encompassing factors such as government interventions and disruptions to non-COVID care [30,40,41]. While excess mortality provides a general understanding, it is equally important to examine changes in cause-specific mortality during the pandemic. This examination enables the development of targeted strategies to mitigate similar impacts in the future. Notably, the pandemic may have indirectly led to increases in causes of death such as heart disease, diabetes, and Alzheimer's disease [38]. In terms of non-natural causes of death, a substantial increase was found in accidental drug-related fatalities during all stages of the lockdown in Ontario, while homicide or suicide rates experienced only moderate changes [11]. Nevertheless, collecting consistent cause-specific mortality data based on death certificates can be challenging [14,17,42]. Our analysis focuses on a nationally curated dataset comprising monthly death counts in Italy from 2015 to 2020, categorized according to the International Classification of Diseases 10th Revision (ICD-10). The dataset is high-dimensional and sparse in nature, which motivates the development of new methods to uncover the underlying relationships during this period.

The Poisson regression model provides a solid foundation for modeling count data [13]. Researchers have also proposed variants of Poisson regression such as overdispersed Poisson regression and zero-inflated Poisson regression with random effects to account for important data features [8,19,27]. However, in practice, linear relationships between response and covariates are typically assumed. Furthermore, the limited number of observed covariates can hinder a comprehensive understanding of the data. In light of this, we construct additional model components to account for the Poisson residuals. The high-dimensional and sparse residuals are organized as a multi-way tensor, which allows for the utilization of various decomposition techniques to achieve dimension reduction.

Theoretical and practical advantages of these techniques, including canonical polyadic (CP) decomposition, Tucker decomposition, and higher-order singular value decomposition (HOSVD), have been extensively studied [5,10,21,26]. They reduce the dimensionality of the parameter space and uncover latent structures in a stable and unique manner under mild conditions. Consequently, the interpretability of the results is enhanced and the efficiency in both data storage and computation is improved. The most common application of these tensor decomposition techniques appears in the context of tensor regression, either as scalar-on-tensor regression or tensor-on-scalar regression. In scalar-on-tensor regression, a Bayesian approach that utilizes PARAFAC decomposition and imposes a novel class of multiway shrinkage priors is developed and illustrated in a neuroimaging application [16]. For tensor-on-scalar regression, multilinear algebra techniques and a set of tensor regression approaches have been combined to model the variational patterns of point clouds and link them to process variables [43]. Beyond tensor regression, these techniques can be directly applied to data. For instance, a Tucker product for dimensionality reduction within a general multilinear tensor regression framework has been proposed for analyzing longitudinal relational data [22].

Different from tensor regression literature, our proposed method aligns more closely with work assuming lower-dimensional tensor structure on Poisson count data. CP decomposition and Tucker decomposition have been implemented on dyadic event counts to achieve dimension reduction and reliable statistical inference [35,36]. A content request prediction algorithm was proposed that employed tensor train decomposition [29]. Motivated by existing literature, we combine Poisson regression with tensor train decomposition and focus on explanatory analysis of the data. The train decomposition is specifically chosen because it is numerically more stable than the classical CP decomposition method and it provides a more accurate representation of the data as tensor cores in train decomposition follow a hierarchically dependent construction by assumption and this feature aligns naturally with the data structure [6,31]. In addition to improved interpretability, the method can be easily scaled to high-dimensional situations, provided that the tensor ranks are kept at a moderate size.

Our primary objective is to understand the effects of covariates, particularly government lockdown policies during the pandemic, on the mortality rates of various causes of death through the Poisson regression component. Additionally, we aim to uncover further information by inferring latent spaces using tensor specification and tensor train decomposition. For instance, the proposed method enables us to cluster Italian regions based on their dynamic mortality patterns over the observed time window. These clusters have different weights on latent classes characterized by mortality trajectories. A high weight indicates that the corresponding latent class plays an important role in defining the cluster, while a low weight suggests the latent class is irrelevant. Temporal evolution of mortality rates in each latent class also reveals interactions between causes of mortality, such as COVID-19 deaths and other infectious diseases, which Poisson regression alone may not adequately capture. More detailed inferences on latent spaces induced by the proposed method are elaborated in Section 5.3. Our inferences are made within a Bayesian framework, where we impose priors on model parameters. Given our focus on explanatory analysis rather than predictive performance, we carefully specify the priors and select a set of prior hyperparameters to avoid inherent issues related to unidentifiability in general latent factor models. To draw posterior samples, we employ an adaptive Metropolis within Gibbs algorithm.

The rest of the paper is organized as follows. In Section 2, we formulate the model and elucidate how to obtain dimension reduction via tensor train decomposition. In Section 3, we describe the prior specification and the Markov chain Monte Carlo (MCMC) algorithm for posterior inference. Results of simulation studies and real data application are shown in Sections 4 and 5, respectively. Finally, Section 6 provides some concluding remarks and future directions.

## 2. BPRTTD model for count data

In this section we will introduce a Poisson regression model with a tensor train decomposition to capture a low-dimensional structural component in the unexplained Poisson variation. In general, an *M*-dimensional tensor A of size $Q_{1}\times Q_{2}\times \cdots \times Q_{M-1}\times Q_{M}$ is said to admit a train decomposition if entries $a_{q_{1},q_{2},\ldots ,q_{M-1},q_{M}}$ of A can be expressed as the sum of $R_{1}R_{2}\cdots R_{M-1}$ terms such that

$$a_{q_{1},q_{2},\ldots ,q_{M-1},q_{M}}=\sum_{r_{1}=1}^{R_{1}}\sum_{r_{2}=1}^{R_{2}}\cdots \sum_{r_{M-1}=1}^{R_{M-1}}g_{q_{1},r_{1}}^{\left(1\right)}g_{q_{2},r_{1},r_{2}}^{\left(2\right)}\cdots g_{q_{M-1},r_{M-2},r_{M-1}}^{\left(M-1\right)}g_{q_{M},r_{M-1}}^{\left(M\right)}.$$

We call $g_{\cdot ,\cdot }^{\left(1\right)},g_{\cdot ,\cdot ,\cdot }^{\left(2\right)},\ldots ,g_{\cdot ,\cdot }^{\left(M\right)}$ tensor train cores and $R_{1},R_{2},\ldots ,R_{M-1}$ tensor train ranks. Similar to the core tensor in Tucker decomposition, $g_{\cdot ,\cdot }^{\left(1\right)},g_{\cdot ,\cdot ,\cdot }^{\left(2\right)},\ldots ,g_{\cdot ,\cdot }^{\left(M\right)}$ provide insights concerning the relationships among tensor dimensions. For instance, the first tensor margin and the second tensor margin are linked by $R_{1}$ latent classes, like the railway coupling that is located at each end of a rail vehicle and connects them together. Tensor train decomposition also facilitates dimension reduction. The dimensions of the tensor train cores are $Q_{1}R_{1}$, $Q_{2}R_{1}R_{2},\ldots ,Q_{M}R_{M-1}$ respectively, which effectively reduces the dimension $Q_{1}Q_{2}\ldots Q_{M-1}Q_{M}$ of tensor A to the dimension $Q_{1}R_{1}+Q_{2}R_{1}R_{2}+\cdots +Q_{M}R_{M-1}$ of the subspace. Several algorithms such as tensor train-singular value decomposition (TT-SVD) algorithm and Tucker-2 algorithm have been proposed for tensor train approximation [5,31]. It is essential to note that the order of dimensions in the tensor matters. The decomposition is performed sequentially from the first dimension $g_{q_{1},r_{1}}^{\left(1\right)}$ to the last one $g_{q_{M},r_{M-1}}^{\left(M\right)}$, with tensor train cores of a particular dimension depending on the cores of its preceding dimension. Therefore, arranging the data in a suitable structure is crucial for meaningful decomposition. Tensor train decomposition offers several theoretical advantages. It encompasses specific tensor decompositions like the canonical polyadic (CP) decomposition and the Tucker decomposition [5,45]. When $R_{1}=R_{2}=\cdots =R_{M-1}=R,g_{q_{m},r_{1},r_{2}}^{\left(m\right)}=0$ for any m=2,…,M−1 and $r_{1}\neq r_{2}$, a CP decomposition is represented in terms of the tensor train decomposition. It can also be shown that there exists an algebraic equivalence between the Tucker and the tensor train decomposition [45]. Despite being interpretable, the CP format and the Tucker format have several disadvantages. For example, the set of tensors of a fixed CP rank is not closed and the computational cost of applying Tucker decomposition grows exponentially fast with the tensor order [3]. In contrast, tensor train decomposition remains stable and straightforward, enabling the summarization of high-dimensional data with a limited number of latent variables. As a consequence, the interpretation of results in practical applications becomes more accessible [31]. Recognizing these merits, we value the tensor train decomposition and incorporate it into our proposed model, which is formulated below.

Suppose the observed count data can be arranged as a three-way discrete-valued tensor $Y_{i,t,k}$ of dimension N×T×K and i=1,…,N,t=1,…,T,k=1,…,K. Additionally, we have information on covariates $x_{i,t,k}\in R^{P}$ and offsets $u_{i,t,k}$. The standard Poisson regression model assumes that $Y_{i,t,k}∼\mathrm{Pois}(u_{i,t,k}\exp(x_{i,t,k}\cdot \beta )),$ describing the relationship between covariates $x_{i,t,k}$ and the dependent variable $Y_{i,t,k}$. However, the regression can potentially fail to account for residual variation, namely Pearson residual or deviance residual that measures the discrepancy of a generalized linear model [28]. This is because only a limited number of variables are included in $x_{i,t,k}$. Even though adding more interaction terms may improve the goodness-of-fit of the Poisson regression model, it quickly becomes unmanageable and hard to interpret if the dimension of $Y_{i,t,k}$ is large.

To address this issue, we propose to combine Poisson regression with the tensor train decomposition technique and form a novel Poisson Regression Tensor Train Decomposition (PRTTD) model that provides an adaptive, low-dimensional alternative, capturing the residual variation in an interpretable way. The model extends Poisson regression with an extra rate parameter $\lambda_{i,t,k}^{*}$

(1) $$Y_{i,t,k}∼\mathrm{Pois}\left(u_{i,t,k}\exp\left(x_{i,t,k}\cdot \beta \right)\lambda_{i,t,k}^{*}\right),$$

and assumes that the rate $\lambda_{i,t,k}^{*}$ can be expressed according to tensor train decomposition such that

$$\begin{array}{rl} \lambda_{i,t,k}^{*} & =\sum_{h_{1}=1}^{H_{1}}\lambda_{i,h_{1}}^{\left(1\right)}\sum_{h_{2}=1}^{H_{2}}\lambda_{t,h_{1},h_{2}}^{\left(2\right)}\lambda_{k,h_{2}}^{\left(3\right)}\\ & =\lambda_{i}^{(1{)}^{\prime }}\Lambda_{t}^{\left(2\right)}\lambda_{k}^{\left(3\right)}, \end{array}$$

where $\lambda_{i}^{\left(1\right)}=(\lambda_{i,1}^{\left(1\right)},\ldots ,\lambda_{i,H_{1}}^{\left(1\right)}{)}^{\prime }\in R_{+}^{H_{1}},\lambda_{k}^{\left(3\right)}=(\lambda_{k,1}^{\left(3\right)},\ldots ,\lambda_{k,H_{2}}^{\left(3\right)}{)}^{\prime }\in R_{+}^{H_{2}}$ and

$$\Lambda_{t}^{\left(2\right)}=\left(\begin{array}{cccc} \lambda_{t,1,1}^{\left(2\right)} & \lambda_{t,1,2}^{\left(2\right)} & \ldots & \lambda_{t,1,H_{2}}^{\left(2\right)}\\ \lambda_{t,2,1}^{\left(2\right)} & \lambda_{t,2,2}^{\left(2\right)} & \ldots & \lambda_{t,2,H_{2}}^{\left(2\right)}\\ \vdots & \vdots & \ldots & \vdots \\ \lambda_{t,H_{1},1}^{\left(2\right)} & \lambda_{t,H_{1},2}^{\left(2\right)} & \ldots & \lambda_{t,H_{1},H_{2}}^{\left(2\right)} \end{array}\right).$$

Here the collection of matrices $\{\lambda_{i}^{\left(1\right)}{\}}_{i=1,\ldots ,N},\{\Lambda_{t}^{\left(2\right)}{\}}_{t=1,\ldots ,T}$ and $\{\lambda_{k}^{\left(3\right)}{\}}_{k=1,\ldots ,K}$ are tensor train cores. $H_{1}$ and $H_{2}$ are tensor train ranks and they control the model complexity. When $H_{1}$ and $H_{2}$ are small relative to *N*, *T* and *K*, this is a parsimonious representation of the rate tensor $\{\lambda_{i,t,k}^{*}{\}}_{i=1,\ldots ,N,t=1,\ldots ,T,k=1,\ldots ,K}$. Initially, the tensor has N⋅T⋅K parameters; whereas the number reduces to $N\cdot H_{1}+T\cdot H_{1}\cdot H_{2}+K\cdot H_{2}$ after using the tensor decomposition representation. In this context, instead of relying on interaction terms common in Poisson regression to understand interactions among covariates, which potentially lead to a prohibitively large number of parameters, we employ the hierarchical decomposition technique to achieve the same goal. In other words, the tensor train decomposition could be interpreted as a way to explore possible complex interactions among the three dimensions using fewer factors. We will see more explicitly the interpretations in Section 5.3.

## 3. Prior specification and posterior inference

Due to the complex nature of the model space, we adopt a Bayesian approach to make inferences. Bayesian methods also provide the necessary uncertainty quantification. We impose gamma priors on $\{\lambda_{i}^{\left(1\right)}{\}}_{i=1,\ldots ,N},\{\Lambda_{t}^{\left(2\right)}{\}}_{t=1,\ldots ,T}$ and $\{\lambda_{k}^{\left(3\right)}{\}}_{k=1,\ldots ,K}$ to exploit the conjugate property of the Poisson parameters; that is

$$\begin{array}{rl} \lambda_{i,h_{1}}^{\left(1\right)} & ∼\mathrm{Ga}(\alpha_{a},\alpha_{b}),\;i=1,\ldots ,N,\;h_{1}=1,\ldots ,H_{1},\\ \lambda_{t,h_{1},h_{2}}^{\left(2\right)} & ∼\mathrm{Ga}(\gamma_{a},\gamma_{b}),\;t=1,\ldots ,T,\;h_{1}=1,\ldots ,H_{1},\;h_{2}=1,\ldots ,H_{2},\\ \lambda_{k,h_{2}}^{\left(3\right)} & ∼\mathrm{Ga}(\epsilon_{a},\epsilon_{b}),\;k=1,\ldots ,K,\;h_{2}=1,\ldots ,H_{2}. \end{array}$$

Posterior inference on these parameters can be obtained by using the Gibbs sampling algorithm conditional on the most recent values of other parameters. As for the Poisson regression coefficients β, we follow the literature and assume zero-mean normal priors such that

$$\beta_{p}∼N(0,\sigma^{2}),\;p=1,\ldots ,P.$$

where $\beta_{p}$ denotes the *p*-th element of the vector β. This completes the prior specification for the BPRTTD model. Since normal priors on β are not conjugate, we sample β using an adaptive Metropolis-Hastings step that learns the posterior correlation between parameters [33]. We outline the MCMC algorithm below.

### 3.1. Adaptive metropolis within Gibbs sampler

We employ a Gibbs sampler for $\lambda_{i,h_{1}},\lambda_{t,h_{1},h_{2}}$ and $\lambda_{k,h_{2}}$ given the Poisson regression coefficients β. The Gibbs sampling algorithm augments the state space with the variable $Y_{i,t,k}^{h_{1},h_{2}}$ such that

(2) $$Y_{i,t,k}^{h_{1},h_{2}}∼\mathrm{Pois}\left(u_{i,t,k}\exp\left(x_{i,t,k}\cdot \beta \right)\lambda_{i,h_{1}}^{\left(1\right)}\lambda_{t,h_{1},h_{2}}^{\left(2\right)}\lambda_{k,h_{2}}^{\left(3\right)}\right).$$

Utilizing the closure under addition property of Poisson random variables, (2) implies that

$$Y_{i,t,k}=\sum_{h_{1}=1}^{H_{1}}\sum_{h_{2}=1}^{H_{2}}Y_{i,t,k}^{h_{1},h_{2}}.$$

To draw $Y_{i,t,k}^{h_{1},h_{2}}$ conditional on $Y_{i,t,k}$ and $\lambda_{i,h_{1}}^{\left(1\right)},\lambda_{t,h_{1},h_{2}}^{\left(2\right)},\lambda_{k,h_{2}}^{\left(3\right)}$, it suffices to note the relationship between the Poisson random variable and the Multinomial random variable, i.e.

$$\left(Y_{i,t,k}^{1,1},Y_{i,t,k}^{1,2},\ldots ,Y_{i,t,k}^{H_{1},H_{2}}\right)∼\mathrm{Multi}\left(Y_{i,t,k},\left(\pi_{i,t,k}^{1,1},\pi_{i,t,k}^{1,2},\ldots ,\pi_{i,t,k}^{H_{1},H_{2}}\right)\right)$$

with $\pi_{i,t,k}^{h_{1},h_{2}}=\lambda_{i,h_{1}}^{\left(1\right)}\lambda_{t,h_{1},h_{2}}^{\left(2\right)}\lambda_{k,h_{2}}^{\left(3\right)}/\sum_{h_{1}=1}^{H_{1}}\sum_{h_{2}=1}^{H_{2}}\lambda_{i,h_{1}}^{\left(1\right)}\lambda_{t,h_{1},h_{2}}^{\left(2\right)}\lambda_{k,h_{2}}^{\left(3\right)}$. Other useful latent quantities for the Gibbs sampler are

$$\begin{array}{rl} Y_{i,\cdot ,\cdot }^{h_{1},\cdot } & =\sum_{t=1}^{T}\sum_{k=1}^{K}\sum_{h_{2}=1}^{H_{2}}Y_{i,t,k}^{h_{1},h_{2}}\\ & ∼\mathrm{Pois}\left(\lambda_{i,h_{1}}^{\left(1\right)}u_{i,t,k}\exp\left(x_{i,t,k}\cdot \beta \right)\sum_{t=1}^{T}\sum_{k=1}^{K}\sum_{h_{2}=1}^{H_{2}}\lambda_{t,h_{1},h_{2}}^{\left(2\right)}\lambda_{k,h_{2}}^{\left(3\right)}\right),\\ Y_{\cdot ,t,\cdot }^{h_{1},h_{2}} & =\sum_{i=1}^{N}\sum_{k=1}^{K}Y_{i,t,k}^{h_{1},h_{2}}\\ & ∼\mathrm{Pois}\left(\lambda_{t,h_{1},h_{2}}^{\left(2\right)}u_{i,t,k}\exp\left(x_{i,t,k}\cdot \beta \right)\sum_{i=1}^{N}\sum_{k=1}^{K}\lambda_{i,h_{1}}^{\left(1\right)}\lambda_{k,h_{2}}^{\left(3\right)}\right),\\ Y_{\cdot ,\cdot ,k}^{\cdot ,h_{2}} & =\sum_{i=1}^{N}\sum_{t=1}^{T}\sum_{h_{1}=1}^{H_{1}}Y_{i,t,k}^{h_{1},h_{2}}\\ & ∼\mathrm{Pois}\left(\lambda_{k,h_{2}}^{\left(3\right)}u_{i,t,k}\exp\left(x_{i,t,k}\cdot \beta \right)\sum_{i=1}^{N}\sum_{t=1}^{T}\sum_{h_{1}=1}^{H_{1}}\lambda_{i,h_{1}}^{\left(1\right)}\lambda_{t,h_{1},h_{2}}^{\left(2\right)}\right). \end{array}$$

With these three auxiliary variables, it is easy to derive the full conditional distributions. To update $\lambda_{i,h_{1}}$, we draw samples from

$$\lambda_{i,h_{1}}|\cdot ∼\mathrm{Ga}\left(\alpha_{a}+Y_{i,\cdot ,\cdot }^{h_{1},\cdot },\alpha_{b}+u_{i,t,k}\exp\left(x_{i,t,k}\cdot \beta \right)\sum_{t=1}^{T}\sum_{k=1}^{K}\sum_{h_{2}=1}^{H_{2}}\lambda_{t,h_{1},h_{2}}^{\left(2\right)}\lambda_{k,h_{2}}^{\left(3\right)}\right).$$

Similarly for $\lambda_{t,h_{1},h_{2}}$ and $\lambda_{k,h_{2}}$, the full conditional distributions are

$$\begin{array}{rl} & \lambda_{t,h_{1},h_{2}}|\cdot ∼\mathrm{Ga}\left(\gamma_{a}+Y_{\cdot ,t,\cdot }^{h_{1},h_{2}},\gamma_{b}+u_{i,t,k}\exp\left(x_{i,t,k}\cdot \beta \right)\sum_{i=1}^{N}\sum_{k=1}^{K}\lambda_{i,h_{1}}^{\left(1\right)}\lambda_{k,h_{2}}^{\left(3\right)}\right)\\ & \lambda_{k,h_{2}}|\cdot ∼\mathrm{Ga}\left(\epsilon_{a}+Y_{\cdot ,\cdot ,k}^{\cdot ,h_{2}},\epsilon_{b}+u_{i,t,k}\exp\left(x_{i,t,k}\cdot \beta \right)\sum_{i=1}^{N}\sum_{t=1}^{T}\sum_{h_{1}=1}^{H_{1}}\lambda_{i,h_{1}}^{\left(1\right)}\lambda_{t,h_{1},h_{2}}^{\left(2\right)}\right). \end{array}$$

After updating $\lambda_{i,h_{1}},\lambda_{t,h_{1},h_{2}}$ and $\lambda_{k,h_{2}}$ at each iteration, β is sampled using an adaptive Metropolis-Hastings step [33] with *n*-step proposal distribution

$$Q_{n}\left(\beta ,\cdot \right)=(1-p)N\left(\beta ,(2.38{)}^{2}\Sigma_{n}/d\right)+pN\left(\beta ,(0.1{)}^{2}\Sigma /d\right),$$

where *p* is a small constant between 0 and 1, $\Sigma_{n}$ is an empirical estimate of the covariance matrix of the target posterior distribution based on the run so far and *d* is the dimension of β. Σ is a fixed covariance matrix and we take it to be the GLM estimate of the Poisson regression covariance matrix for efficiency.

## 4. Simulation studies

We have carried out two simulation studies to validate the BPRTTD model and its associated posterior sampling algorithm. The first study involves the creation of artificial parameters, which are then utilized to generate Poisson observations. We set *N* = 20, *T* = 20, *K* = 20, and the rank of tensor train decomposition $H_{1}=H_{2}=5$. We incorporate in the model one intercept and *P* = 5 covariates, whose regression coefficients β are sampled from a centered normal distribution with a variance of 0.1. $\lambda_{i,h_{1}}^{\left(1\right)}$ is generated from a gamma distribution with parameters shape equal to 1 and rate equal to 2.8. Similarly, $\lambda_{t,h_{1},h_{2}}^{\left(2\right)}$ and $\lambda_{k,h_{2}}^{\left(3\right)}$ are simulated from the same gamma distribution. After fixing the parameter values, we generate covariates $x_{i,t,k}$ from a standard normal distribution and the offset $u_{i,t,k}$ from a gamma distribution with shape and rate set to 5 and 1 respectively. We repeat this simulation process 100 times, generating observed data $Y_{i,t,k}$ for each repetition. Finally, we fit the BPRTTD model to the simulated data. At this stage, we assume that the true latent dimension $H_{1}$ and $H_{2}$ are known, and set the parameters of the prior distribution as follows: $\alpha_{a}=1,\alpha_{b}=1,\beta_{a}=1,\beta_{b}=2,\epsilon_{a}=1,\epsilon_{b}=1$. The prior variance of β is 0.1. The probability of sampling from the rescaled empirical normal distribution in the proposal of the adaptive Metropolis-Hastings algorithm to update regression coefficients is *p* = 0.95. We run the Markov chain Monte Carlo (MCMC) simulation for 10,000 iterations, discarding the first 3000 iterations as burn-in. Partial comparison between true β and the estimated ones is shown in Table 1. The method is able to correctly estimate the true regression parameters β for both the intercept and the covariates. Note that the estimates associated with simulated covariates, $x_{i,t,k}$, have smaller standard deviations (estimated over 100 Monte Carlo repetitions) than the standard deviations of the intercepts. This is due to the identifiability issue associated with the intercept and $\lambda_{i,h_{1}}^{\left(1\right)},\lambda_{t,h_{1},h_{2}}^{\left(2\right)},\lambda_{k,h_{2}}^{\left(3\right)}$ inherent to the BPRTTD model as these parameters multiply and contribute to the Poisson rate. The careful choice of prior parameters helps overcome the identifiability problem and facilitates our goal to interpret factors $\lambda_{i,h_{1}}^{\left(1\right)},\lambda_{t,h_{1},h_{2}}^{\left(2\right)},\lambda_{k,h_{2}}^{\left(3\right)}$. The remaining results are reported in the Supplementary Material.

**Table 1.** Comparison between true β and the estimated $\hat{\beta_{1}}$ from the BPRTTD model and $\hat{\beta_{2}}$ from the BPRCPD model in terms of posterior mean. The first column shows the estimated intercpet and the remaining columns show the estimated coefficient associated with covariates. Averages and standard deviations (in parentheses) of the posterior means over 100 repetitions are reported.

β	−0.0387	0.1747	0.1103	0.1137	0.2840	−0.6080
$\hat{\beta_{1}}$	0.0078	0.1753	0.1100	0.1133	0.2841	−0.6074
	(0.0342)	(0.0049)	(0.0038)	(0.0042)	(0.0044)	(0.0048)
$\hat{\beta_{2}}$	0.1079	0.1748	0.1098	0.1132	0.2839	−0.6073
	(0.0517)	(0.0057)	(0.0047)	(0.0044)	(0.0049)	(0.0055)

In addition, we also compare our method with another Bayesian Poisson Regression CP decomposition (BPRCPD) model where $\lambda_{i,t,k}^{*}$ is assumed to admit a CP decomposition with *H* = 6. Gamma priors with the same rate and shape parameters are imposed and 10,000 MCMC iterations are run as for estimating the proposed BPRTTD model. Comparisons are displayed in Table 1. We notice that both methods are able to recover the true regression coefficients; however, the estimated BPRTTD coefficients have slightly smaller standard deviations, indicating that correct model specification does help reduce the estimation uncertainty in this simulation setup.

It is worth noting, however, that the dimension of the simulated data in this study is significantly smaller than what is typically encountered in real-world applications. This choice is deliberate, allowing us to perform multiple simulation repetitions. An additional constraint of this study is that the true parameters, represented as β, are drawn from an arbitrary normal distribution. Moreover, the parameters $\lambda_{i}^{\left(1\right)},i=1,\ldots ,N,\Lambda_{t}^{\left(2\right)},t=1,\ldots ,T,\lambda_{k}^{\left(3\right)},k=1,\ldots ,K$ are simulated from a gamma distribution with artificial shape and rate values. This approach may not accurately mirror a typical real-world data scenario.

To address these limitations, we designed a second simulation study. This study derives true parameters from real data, based on the BPRTTD model specification. More specifically, we use the real data and apply the proposed BPRTTD method to obtain estimates of the parameters $\beta ,\lambda_{i,h_{1}}^{\left(1\right)},\lambda_{t,h_{1},h_{2}}^{\left(2\right)},\lambda_{k,h_{2}}^{\left(3\right)}$. Once the estimates have been acquired, we treat them as true parameters and simulate Poisson observations as per the (1) specification. In this step, offsets $u_{i,t,k}$ are generated from a gamma distribution with shape parameter equal to $10^{6}$ and rate of 1.

We then apply our methodology to the simulated data, aiming to recover the true parameters within this high-dimensional and more realistic context. A summary of the absolute percentage error (APE) between the true parameters and their posterior mean estimates is presented in Table 2. The results indicate that, using our method, at least 75% of the parameters $\beta ,\lambda_{i,h_{1}}^{\left(1\right)},\lambda_{t,h_{1},h_{2}}^{\left(2\right)}$, and $\lambda_{k,h_{2}}^{\left(3\right)}$ in the BPRTTD model are recovered with less than a 40% deviation from their true values.

**Table 2.** Summary statistics of the APE between true parameters and the estimated posterior means in the second simulation study.

	Min.	1st Qu.	Median	Mean	3rd Qu.	Max.
$|\hat{\beta }-\beta |/|\beta |$	0.0005	0.0367	0.0978	0.2316	0.2667	4.4938
$|{\hat{\lambda }}_{i,h_{1}}^{\left(1\right)}-\lambda_{i,h_{1}}^{\left(1\right)}|/|\lambda_{i,h_{1}}^{\left(1\right)}|$	0.0000	0.0095	0.0228	0.0331	0.0448	0.6248
$|{\hat{\lambda }}_{t,h_{1},h_{2}}^{\left(2\right)}-\lambda_{t,h_{1},h_{2}}^{\left(2\right)}|/|\lambda_{t,h_{1},h_{2}}^{\left(2\right)}|$	0.0000	0.0083	0.0196	0.0280	0.0371	0.3162
$|{\hat{\lambda }}_{k,h_{2}}^{\left(3\right)}-\lambda_{k,h_{2}}^{\left(3\right)}|/|\lambda_{k,h_{2}}^{\left(3\right)}|$	0.0005	0.0282	0.0671	0.1466	0.1372	2.1705

## 5. Causes of death in Italy from 2015 to 2020

We apply the BPRTTD model to Italian mortality data in an effort to comprehend the evolving mortality patterns of COVID-19 and other causes of death, both prior to and during the pandemic. These data include provisional monthly death counts derived from the analysis of death certificates that doctors compiled for all Italian deaths between January 2015 and December 2020. These counts correspond to *K* = 18 distinct causes of death and span *T* = 72 monthly death counts. Furthermore, the death counts are grouped into *N* = 420 strata, defined by 10 age groups, two sexes, and 21 Italian regions. Consequently, we observe $Y_{i,t,k}$ for i=1,…,N,t=1,…,T,k=1,…,K, with a total of 544,320 observations arranged in a N×T×K multiway array. The 544,320 observations include a total of 288,591 non-zero counts, and these non-zero counts range from 1 to 1140 with a mean 13.6192 and a standard deviation 30.8174.

Along with death counts $Y_{i,t,k}$, we incorporate covariates $x_{i,t,k}$. A variable of primary interest in our analysis is the Italian Stringency Index (ISI), proposed and developed similarly to the Oxford Stringency Index (OSI) by [7,18]. This variable quantifies the non-pharmaceutical interventions employed by Italian authorities to combat the COVID-19 pandemic, providing insight at both national and regional levels. Regional stringency indices are particularly relevant given that our mortality counts are region-based. We explore potential interactions between the ISI and various causes of death, in light of literature suggesting the pandemic may have distinct impacts on different mortality causes. We also consider two other groups of covariates: interactions between age groups and causes of death, and interactions between age groups and sex. Age and sex are recognized risk factors for numerous causes of death, with distinct mortality patterns often apparent between males and females across various age brackets. These interaction terms contribute to a total of 208-dimensional covariates, $x_{i,t,k}$, in the model. The offset, $u_{i,t,k}$ includes the number of days in each month, the monthly aggregated COVID-19 cases, and the population for all other causes of death. For external causes such as trauma and poisoning, we consider an additional offset reflecting mobility levels. We leverage the Google COVID-19 Community Mobility Reports as an indicator of this. By integrating the mobility offset into the Poisson rate, we can model changes in the mortality rate of external causes of death per fixed mobility unit.

The remaining Poisson rate $\lambda_{i,t,k}^{*}$, not accounted for by the regression component, is assumed to admit tensor train decomposition with rank $H_{1}=6$ and $H_{2}=6$. The intuition behind applying the tensor train decomposition to the data is that we assume there is a lower-dimensional approximation in the 420 demographic strata in terms of $H_{1}$ latent classes. On top of that, each latent class is further decomposed into $H_{2}$ latent classes characterized by time-varying mortality patterns of 18 causes of death over 72 months. This hierarchical structure assumption naturally coincides with tensor train decomposition. These values were determined after testing various combinations of $H_{1}$ and $H_{2}$ over grids defined by $H_{1}=5,6,7,8$ and $H_{2}=5,6,7,8$. The chosen values strike a balance between reasonable model fitting and model complexity. The Gamma priors on $\lambda_{i,h_{1}}^{\left(1\right)},\lambda_{t,h_{1},h_{2}}^{\left(2\right)},\lambda_{k,h_{2}}^{\left(3\right)}$ have parameters such that $\alpha_{b}=20,\alpha_{a}=\sqrt{1/(H1*H2)}*\alpha_{b},\gamma_{b}=20,\gamma_{a}=\sqrt{1/(H1*H2)}*\gamma_{b},\epsilon_{a}=200,\epsilon_{b}=200$. For the Poisson regression coefficients β, we impose centered normal priors with variance equal to 2. The MCMC iterations are 40,000. Finally, we add some remarks on the computational efficiency of the algorithm. Theoretically, the algorithm complexity is linear in $H_{1}*H_{2}$. For instance, when $H_{1}=6,H_{2}=6$, it takes roughly 15 hours to draw 40,000 MCMC samples. The simulation time is reduced to 12 hours for $H_{1}=5,H_{2}=6$ and $H_{1}=6,H_{2}=5$. The reported computational times are observed on macOS Monterey with a 2 GHz Quad-Core Intel Core i5 processor and 16 GB 3733 MHz LPDDR4X memory.

### 5.1. Improvement of BPRTTD model over Poisson regression

**Figure 1.**
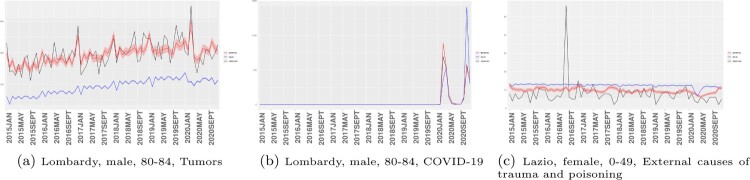
Death counts of selected demographic groups and causes of death from January 2015 to December 2020. The black line represents the observed trajectory $Y_{i,t,k},t=1,\ldots ,T$ for fixed *i* and *k*; red and blue lines represent BPRTTD fitted values and GLM fitted values respectively. Shaded areas correspond to 95% credible intervals for BPRTTD predictions and 95% confidence interval for GLM predictions. (a) Lombardy, male, 80–84, Tumors. (b) Lombardy, male, 80–84, COVID-19 and (c) Lazio, female, 0–49, External causes of trauma and poisoning.

First, we highlight the contribution of the additional tensor decomposition component to fitting the Poisson rate. Figure 1 depicts how our method refines the GLM estimates, allowing them to more accurately recover the observed fluctuations in death counts $Y_{i,t,k}$. In selected trajectories, the tensor decomposition component corrects GLM estimates to more closely align with observed trajectories. As an example, the GLM-predicted death counts for males aged 80–84 who resided in Lombardy and died from tumors are consistently lower than observed counts. This is not surprising, as the GLM estimates fit using the average of all observations, while Lombardy, the most populated region in Italy, generally records higher death counts. Our method effectively bridges the gap between data and GLM estimates by amplifying the Poisson rates, as demonstrated in Figure 1(a).

When GLM overpredicts, as depicted in Figure 1(b), $\lambda_{i,t,k}^{*}$ serves to reduce the Poisson rate. The tensor decomposition assumption allows for such adjustments to be made in a parsimonious manner. Note that a saturated model would require a total of 544,320 parameters. In contrast, our approach introduces only $N\times H_{1}+T\times H_{1}\times H_{2}+K\times H_{2}=5220$ additional parameters, aside from the 208 coefficients, resulting in a significant improvement in model fitting. This advantage is evident when comparing the log-likelihood of a simple Poisson regression versus our BPRTTD model, which respectively stand at −862910.4 and −731919.9; or when comparing the deviance goodness-of-fit, which are 106666274.0 and 463324.2 for a simple Poisson regression and the proposed BPRTTD model, respectively. Even though our approach offers further approximation to observations, it remains robust to outliers or abnormal records. The model specification exploits and leverages information from other observations by introducing commonly shared latent tensor cores. Figure 1(c) illustrates such a scenario, where female mortality counts in the 0–49 age group in Lazio in August 2016 demonstrate a sudden spike, deviating from the normal pattern. The BPRTTD line, shown in red, is not sensitive to such an outlier.

### 5.2. Interpretation of Poisson regression component

In this section, we aim to answer the question of how other causes of death are affected by government intervention policies. Based on whether the 95% credible intervals of each coefficient are above 0, below 0, or contain 0, we infer three types of responses: positive, negative, and no effects. Mortality counts are positively associated with ISI in the following death categories: diseases of the blood and hematopoietic organs and disorders of the immune system; endocrine, nutritional, and metabolic diseases; psychic and behavioral disorders; diseases of the nervous system and sense organs; diseases of the respiratory system; diseases of the musculoskeletal system and connective tissue; diseases of the genitourinary system, symptoms, signs, abnormal results, and ill-defined causes; and external causes of trauma and poisoning. The positive relationship between ISI and psychic disorders, affecting both psychiatric patients and the general population, is well documented in the literature [20,34]. While many studies report increasing levels of anxiety and acute stress disorders, our findings provide new evidence that these factors actually translate to elevated mortality rates from psychic and behavioral disorders. Another positive relationship of interest is between ISI and mortality due to respiratory system diseases. Despite studies suggesting a decline in respiratory disease incidences due to public precautionary measures [2,23,24], we find that the mortality rate from respiratory diseases increases during the COVID-19 lockdown. Factors like disruption to routine care and misclassification of cause of death in the early pandemic can explain this increase. For mortality due to external causes of trauma and poisoning, we discover an upward trend as more intense lockdown measures are enforced, contradicting expectations. After adjusting for the negative effect of lockdown on population mobility, we attribute this to reduced or delayed access to healthcare caused by government intervention policies.

Negative correlations appear in infectious and parasitic diseases; tumors; diseases of the circulatory system; diseases of the digestive system; complications of pregnancy, childbirth, and the puerperium; morbid conditions that originate in the perinatal period; and congenital malformations and chromosomal anomalies. It has been observed that infectious and parasitic diseases caused less mortality when government interventions were stricter [9]. One explanation for the decrease in tumor mortality rate is the harvesting effect or mortality displacement [25,37], which refers to the phenomenon where individuals who are already vulnerable, in this case, tumor patients, experience accelerated deaths during the COVID-19 lockdown intervention, leading to a temporary decline in tumor mortality rates. However, this decline is expected to be followed by a period of increased mortality as those who would have died during the intervention succumb in the subsequent period. Only one category, diseases of the skin and subcutaneous tissue, exhibits no statistically significant relationship with ISI. We also observe the effect of sex and age on the hazard rates. In general, an older population is associated with higher mortality in almost all types of death causes, and men are more likely to die than women in the same age group. The exception is with tumors, where men from certain younger age groups present higher mortality rates compared to women from older age groups. It is also counterintuitive to find a positive relation between age and the mortality rate due to external causes of trauma and poisoning. Although the absolute death counts decrease with age, the mortality rates per population unit increase, suggesting that these external causes become more threatening as people age. We present predicted mortality rates for selected causes of death in the Supplementary Material.

### 5.3. Interpretation of latent tensor cores

Three blocks of latent tensor cores are introduced in the BPRTTD model, and they are arranged in a dependent structure; that is, each latent class $\lambda_{i,h_{1}}^{\left(1\right)}$ is characterized by different $\lambda_{t,h_{1},h_{2}}^{\left(2\right)}$, and furthermore, $h_{2}$-specific $\lambda_{k,h_{2}}^{\left(3\right)}$. This structure necessitates a systematic approach to the interpretation of latent parameters.

The first block of tensor cores $\lambda_{i,h_{1}}^{\left(1\right)}$ allocates demographic groups, defined by Italian regions, sex, and age groups, into $H_{1}$ latent classes. The posterior mean estimates of $\lambda_{i,h_{1}}^{\left(1\right)}$ are displayed in Table 3, with values above the mean $\alpha_{a}/\alpha_{b}$ of the Gamma prior distribution highlighted in red. As these estimates reveal differential local effects of higher-order interactions between covariates on mortality rates unaccounted for by Poisson regression, they provide key insights into the unique demographic mortality patterns.

The latent classes $h_{1}=1$ and $h_{1}=4$ predominantly represent female and male mortality patterns, respectively, albeit with notable geographical dependencies. For instance, the majority of female age groups, except older females (age group 85+) from southern Italy (Molise, Campania, Apulia, Basilicata, Calabria, Sicily), exhibit increased weights in latent class $h_{1}=1$, as demonstrated in Table 3(a). These same older southern Italian women share similar mortality patterns with nearly all male groups, excluding those in northern Italy (Piemonte, Valle d'Aosta, Lombardy, Veneto, Friuli-Venezia Giulia, Emilia-Romagna), as illustrated in Table 3(d). Interestingly, latent class $h_{1}=6$ indicates a shared mortality pattern between old males and young females.

**Table 3.** Posterior mean estimates of $\lambda_{i,h_{1}}^{\left(1\right)},i=1,\ldots ,N,h_{1}=1,\ldots ,H_{1}$, from the BPRTTD model. Red-colored numbers indicate that the estimates are higher than the prior mean.

	Male 00–49	50–59	60–64	65–69	70–74	75–79	80–84	85–89	90–94	95+	Female 00–49	50–59	60–64	65–69	70–74	75–79	80–84	85–89	90–94	95+
(a) ${\hat{\lambda }}_{i,1}^{\left(1\right)}$																				
Piedmont	0.2176	0.1779	0.2456	0.1583	0.1277	0.1419	0.1351	0.1061	0.0799	0.0431	0.1312	0.2436	0.2442	0.4634	0.4536	0.4853	0.4126	0.3386	0.2555	0.2102
Aosta Valley	0.0926	0.1480	0.1575	0.2142	0.1906	0.1744	0.3431	0.3038	0.1781	0.1307	0.1568	0.1502	0.1883	0.1688	0.2560	0.3198	0.4968	0.4399	0.5008	0.4501
Lombardy	0.1580	0.1784	0.0874	0.0688	0.0531	0.0427	0.0786	0.0836	0.0991	0.0644	0.0989	0.1233	0.2349	0.3042	0.3308	0.3454	0.3565	0.3446	0.3095	0.3090
Veneto	0.2859	0.2770	0.3613	0.2588	0.3013	0.2781	0.2858	0.2892	0.2818	0.2949	0.1447	0.2803	0.3251	0.4609	0.4846	0.5100	0.5346	0.5167	0.4924	0.4948
Friuli-Venezia Giulia	0.1308	0.2867	0.3383	0.2717	0.2647	0.1368	0.1372	0.1331	0.1502	0.1148	0.0800	0.1073	0.2918	0.3231	0.4759	0.3324	0.2955	0.2565	0.1991	0.1577
Liguria	0.1206	0.1908	0.1213	0.1934	0.0668	0.0908	0.0809	0.0654	0.0774	0.0998	0.0862	0.1138	0.1622	0.2711	0.3286	0.4116	0.4099	0.3926	0.2597	0.2784
Emilia-Romagna	0.1650	0.2920	0.2089	0.2036	0.1461	0.1841	0.1742	0.1811	0.1909	0.1767	0.1261	0.2044	0.3586	0.3934	0.4405	0.4047	0.4522	0.4153	0.3806	0.3645
Tuscany	0.1949	0.2870	0.2403	0.1431	0.1527	0.1582	0.1370	0.1098	0.0553	0.0454	0.0762	0.1685	0.3080	0.3947	0.4376	0.4963	0.4255	0.3874	0.3217	0.2066
Umbria	0.1951	0.1352	0.0933	0.1233	0.1003	0.1466	0.1237	0.1010	0.1306	0.1302	0.1203	0.1426	0.2229	0.1926	0.2821	0.3066	0.3960	0.3807	0.3847	0.3464
Marche	0.1834	0.2070	0.1385	0.1205	0.1300	0.1879	0.1814	0.1770	0.1806	0.1553	0.0878	0.1647	0.2547	0.2451	0.3994	0.4099	0.4478	0.5024	0.3929	0.3967
Lazio	0.1070	0.1517	0.0587	0.0650	0.0632	0.0499	0.0421	0.0345	0.0110	0.0173	0.0789	0.1201	0.2639	0.2836	0.3318	0.3118	0.2642	0.2366	0.1886	0.1320
Abruzzo	0.1707	0.1742	0.1439	0.1405	0.1242	0.0753	0.1298	0.1322	0.1270	0.1139	0.1865	0.1763	0.3162	0.3039	0.3419	0.3628	0.3815	0.3426	0.3122	0.2468
Molise	0.1028	0.1572	0.1334	0.1739	0.1648	0.0944	0.0473	0.0381	0.0579	0.0543	0.1614	0.1546	0.1144	0.2131	0.1831	0.1916	0.1596	0.0704	0.0751	0.0504
Campania	0.0268	0.0282	0.0348	0.0139	0.0123	0.0059	0.0045	0.0041	0.0053	0.0117	0.0143	0.0324	0.1198	0.1600	0.1957	0.1017	0.0957	0.0704	0.0522	0.0347
Apulia	0.2665	0.1652	0.0755	0.1054	0.0980	0.0569	0.0620	0.0539	0.0196	0.0302	0.1233	0.1598	0.3070	0.3111	0.4298	0.3769	0.2997	0.2679	0.2077	0.1095
Basilicata	0.1661	0.1156	0.1079	0.1255	0.0860	0.0578	0.0847	0.0388	0.0381	0.0724	0.1428	0.1165	0.1801	0.1799	0.2696	0.2357	0.2286	0.1318	0.1218	0.1337
Calabria	0.1114	0.1237	0.0686	0.0648	0.0343	0.0416	0.0167	0.0099	0.0133	0.0225	0.0801	0.1475	0.1813	0.2559	0.2840	0.1434	0.0990	0.0750	0.0456	0.0290
Sicily	0.1935	0.0603	0.0631	0.0254	0.0218	0.0104	0.0081	0.0060	0.0067	0.0157	0.0993	0.0879	0.2348	0.3195	0.3650	0.2447	0.1733	0.1254	0.0737	0.0494
Sardinia	0.2822	0.4700	0.2780	0.1726	0.1961	0.2469	0.2395	0.2195	0.2347	0.1007	0.1112	0.2799	0.3253	0.4373	0.5476	0.5361	0.5265	0.4876	0.4780	0.3805
Bolzano	0.0690	0.1217	0.1862	0.2504	0.1110	0.1285	0.1578	0.2115	0.2872	0.1487	0.1268	0.1486	0.2377	0.2467	0.2636	0.4329	0.4002	0.4235	0.4572	0.3867
Trento	0.0894	0.1515	0.1894	0.1618	0.1207	0.1091	0.0845	0.0982	0.0883	0.0906	0.1109	0.1300	0.1230	0.2024	0.2427	0.2028	0.2475	0.2549	0.2873	0.2906
(b) ${\hat{\lambda }}_{i,2}^{\left(1\right)}$																				
Piedmont	0.0089	0.0109	0.0238	0.0316	0.0294	0.0362	0.0907	0.1551	0.2000	0.2607	0.0255	0.0169	0.0426	0.0509	0.1417	0.1882	0.2719	0.3593	0.3935	0.3755
Aosta Valley	0.1243	0.1338	0.1148	0.1229	0.1238	0.0998	0.0977	0.1198	0.1042	0.1258	0.1501	0.0978	0.1301	0.1147	0.1123	0.1395	0.1525	0.0993	0.1654	0.1614
Lombardy	0.0078	0.0167	0.0171	0.0164	0.0122	0.0140	0.0185	0.0361	0.0572	0.0501	0.0112	0.0090	0.0224	0.0140	0.0398	0.0739	0.1339	0.1517	0.1470	0.1315
Veneto	0.0406	0.0634	0.0660	0.1058	0.0936	0.0813	0.0765	0.0459	0.0333	0.0532	0.0290	0.0271	0.0471	0.0581	0.1283	0.1450	0.1768	0.1756	0.1343	0.1047
Friuli-Venezia Giulia	0.1997	0.1918	0.2055	0.1454	0.1678	0.1003	0.0642	0.0251	0.0194	0.0393	0.0978	0.0550	0.1115	0.1115	0.0587	0.1255	0.0946	0.0584	0.0438	0.0336
Liguria	1.0496	0.8761	0.6517	0.5022	0.4272	0.2426	0.1288	0.0758	0.0209	0.0396	0.5202	0.2459	0.2946	0.2830	0.2634	0.2517	0.2261	0.1600	0.1080	0.0862
Emilia-Romagna	0.1693	0.0619	0.0431	0.0487	0.0268	0.0211	0.0251	0.0171	0.0124	0.0171	0.0742	0.0463	0.0395	0.0904	0.0885	0.1099	0.1442	0.1240	0.0758	0.0608
Tuscany	0.0657	0.0921	0.0837	0.0990	0.0426	0.0944	0.0842	0.1455	0.2342	0.2796	0.0446	0.0614	0.0412	0.0917	0.1277	0.1558	0.2549	0.3401	0.3873	0.4166
Umbria	0.0626	0.0476	0.1582	0.1088	0.0492	0.0427	0.0551	0.0917	0.1077	0.1289	0.0799	0.0616	0.1004	0.0723	0.1615	0.0901	0.1418	0.1903	0.2205	0.2788
Marche	0.0155	0.0318	0.0443	0.0314	0.0271	0.0210	0.0143	0.0108	0.0216	0.0265	0.0293	0.0292	0.0520	0.0442	0.0702	0.0394	0.0569	0.0220	0.0184	0.0333
Lazio	0.0871	0.1031	0.0560	0.0563	0.0478	0.0687	0.0280	0.0157	0.0125	0.0113	0.0507	0.0599	0.1159	0.0992	0.1323	0.1450	0.1171	0.0737	0.0561	0.0348
Abruzzo	0.0455	0.0435	0.0706	0.0814	0.0458	0.0269	0.0237	0.0259	0.0240	0.0353	0.0562	0.0638	0.1280	0.0856	0.1385	0.0716	0.0794	0.0630	0.0587	0.0773
Molise	0.1017	0.2080	0.2883	0.1259	0.1463	0.1526	0.1089	0.1199	0.0687	0.1397	0.1706	0.0919	0.1556	0.2198	0.2260	0.2595	0.2090	0.1564	0.1407	0.1911
Campania	1.1864	1.1549	0.9085	0.7452	0.5601	0.4839	0.3232	0.1704	0.0853	0.0300	0.4727	0.5355	0.6787	0.7316	0.7963	0.7399	0.5390	0.3115	0.2015	0.1338
Apulia	0.0184	0.1335	0.1052	0.1585	0.1693	0.1264	0.1167	0.1204	0.1580	0.1115	0.0508	0.0912	0.1361	0.2114	0.3139	0.3634	0.2830	0.2561	0.2530	0.2505
Basilicata	0.2555	0.2788	0.2481	0.1980	0.1216	0.1764	0.0822	0.0323	0.0930	0.0646	0.1770	0.1857	0.1608	0.2017	0.3689	0.3366	0.2542	0.0844	0.1103	0.0872
Calabria	0.3779	0.5199	0.3678	0.3644	0.3479	0.3168	0.3199	0.2713	0.2379	0.1793	0.2462	0.2947	0.4519	0.4499	0.5928	0.6148	0.4710	0.4175	0.3748	0.3053
Sicily	0.2371	0.4084	0.4387	0.4151	0.4585	0.4146	0.4426	0.4081	0.4344	0.3837	0.2718	0.3370	0.3584	0.5116	0.6086	0.7775	0.7962	0.6960	0.6333	0.5496
Sardinia	0.0211	0.0490	0.0566	0.0578	0.0350	0.0558	0.0882	0.1028	0.1998	0.3013	0.0428	0.0424	0.0580	0.0768	0.0678	0.1196	0.2039	0.2144	0.2779	0.3851
Bolzano	0.0290	0.0393	0.0710	0.0737	0.0630	0.0503	0.0340	0.0255	0.0324	0.0775	0.0423	0.0525	0.0650	0.0695	0.0557	0.0462	0.0513	0.0315	0.0263	0.0437
Trento	0.0569	0.0601	0.0806	0.1032	0.0951	0.0911	0.1739	0.0824	0.1175	0.0693	0.0497	0.0605	0.0942	0.1456	0.1120	0.1656	0.1144	0.2025	0.1009	0.1041
(c) ${\hat{\lambda }}_{i,3}^{\left(1\right)}$																				
Piedmont	0.0531	0.1042	0.1188	0.0778	0.1016	0.1337	0.1417	0.1140	0.0777	0.0238	0.0812	0.0251	0.0347	0.0462	0.0550	0.0696	0.1190	0.2297	0.2887	0.3435
Aosta Valley	0.1128	0.0972	0.1257	0.1640	0.1139	0.1328	0.1712	0.1546	0.0859	0.1105	0.1363	0.1147	0.1237	0.0812	0.1463	0.1624	0.1544	0.2364	0.2585	0.3347
Lombardy	0.0681	0.0398	0.0284	0.0301	0.0445	0.0485	0.0635	0.0297	0.0060	0.0047	0.0415	0.0254	0.0152	0.0138	0.0106	0.0108	0.0232	0.0583	0.0900	0.1469
Veneto	0.0396	0.0567	0.0541	0.0567	0.0655	0.0825	0.0957	0.0829	0.0579	0.0299	0.0566	0.0205	0.0299	0.0139	0.0273	0.0334	0.0607	0.1739	0.2895	0.3717
Friuli-Venezia Giulia	0.1152	0.0636	0.0894	0.0976	0.0876	0.1632	0.1575	0.1511	0.1357	0.1231	0.0669	0.0769	0.0532	0.0415	0.0624	0.1062	0.1882	0.2467	0.4197	0.7280
Liguria	0.0624	0.1124	0.1465	0.1851	0.2059	0.2469	0.2561	0.2771	0.2340	0.0912	0.0712	0.1064	0.1370	0.1253	0.1587	0.1768	0.2315	0.2735	0.4091	0.4193
Emilia-Romagna	0.1023	0.0483	0.0678	0.0700	0.0795	0.0778	0.0985	0.1103	0.0960	0.0831	0.0804	0.0462	0.0468	0.0355	0.0232	0.0408	0.0787	0.1927	0.3211	0.4643
Tuscany	0.1256	0.0381	0.0743	0.0731	0.0939	0.0744	0.0940	0.0887	0.0606	0.0268	0.0558	0.0369	0.0664	0.0303	0.0388	0.0488	0.0872	0.1194	0.1816	0.2499
Umbria	0.1102	0.1265	0.1883	0.1281	0.2568	0.1747	0.2003	0.1395	0.1092	0.0971	0.0914	0.0692	0.1674	0.1072	0.1403	0.1670	0.1606	0.1920	0.1570	0.1775
Marche	0.0874	0.0990	0.1594	0.0760	0.0957	0.1347	0.1042	0.1160	0.1549	0.0425	0.1118	0.0487	0.0971	0.0725	0.0854	0.0935	0.1235	0.1813	0.3082	0.3175
Lazio	0.4921	0.4960	0.4334	0.3473	0.2902	0.2382	0.2260	0.1625	0.1005	0.0376	0.2178	0.2111	0.1866	0.2277	0.1941	0.1626	0.2428	0.2755	0.2632	0.2586
Abruzzo	0.1046	0.1055	0.1220	0.1733	0.1127	0.0742	0.0894	0.0867	0.0395	0.0486	0.0865	0.0881	0.0869	0.0816	0.1050	0.0829	0.0598	0.1089	0.1552	0.1667
Molise	0.1567	0.1963	0.2348	0.2204	0.2147	0.1109	0.1596	0.1409	0.1099	0.1302	0.1698	0.1362	0.1537	0.1680	0.1706	0.0922	0.1472	0.2220	0.2698	0.1558
Campania	0.1231	0.1355	0.1308	0.1247	0.0994	0.0382	0.0102	0.0031	0.0029	0.0047	0.0455	0.0455	0.0444	0.0371	0.0215	0.0122	0.0104	0.0051	0.0062	0.0113
Apulia	0.2046	0.2630	0.2402	0.2365	0.2174	0.1728	0.1556	0.1084	0.0587	0.0455	0.2476	0.1397	0.1654	0.1018	0.1074	0.0750	0.1278	0.1031	0.1648	0.1586
Basilicata	0.2320	0.1932	0.2394	0.2325	0.2024	0.1625	0.1305	0.1923	0.0868	0.1132	0.1070	0.0909	0.2418	0.1201	0.1505	0.1552	0.1497	0.1917	0.2322	0.1563
Calabria	0.1013	0.0914	0.1414	0.0890	0.0626	0.0452	0.0181	0.0251	0.0177	0.0257	0.0938	0.0859	0.0886	0.0702	0.0493	0.0369	0.0354	0.0274	0.0295	0.0496
Sicily	0.3061	0.3217	0.2478	0.2359	0.2623	0.1673	0.0956	0.0617	0.0302	0.0290	0.1088	0.1310	0.1718	0.1878	0.1355	0.0472	0.0617	0.1031	0.1021	0.0645
Sardinia	0.1349	0.2406	0.3515	0.3878	0.2748	0.2502	0.2038	0.1726	0.0864	0.0622	0.1479	0.1231	0.0844	0.1332	0.1125	0.2329	0.1624	0.1975	0.2231	0.1278
Bolzano	0.0505	0.0388	0.0556	0.0258	0.0359	0.0390	0.0341	0.0335	0.0417	0.0435	0.0949	0.0550	0.0547	0.0458	0.0382	0.0325	0.0607	0.0552	0.1585	0.3102
Trento	0.0842	0.0727	0.1164	0.0799	0.1612	0.1404	0.2461	0.3021	0.2375	0.1949	0.0894	0.0852	0.0998	0.0873	0.1482	0.1280	0.2938	0.3026	0.5033	0.5873
(d) ${\hat{\lambda }}_{i,4}^{\left(1\right)}$																				
Piedmont	0.4170	0.4056	0.3023	0.2667	0.2324	0.1878	0.1423	0.1172	0.1091	0.1194	0.0281	0.0316	0.0705	0.0921	0.0543	0.0530	0.0482	0.0137	0.0155	0.0271
Aosta Valley	0.6247	0.4970	0.2679	0.3391	0.2693	0.1550	0.1538	0.0919	0.0781	0.1594	0.1438	0.1072	0.1294	0.1307	0.1547	0.1373	0.0888	0.0841	0.0404	0.0572
Lombardy	0.3005	0.2369	0.1946	0.1430	0.1119	0.0946	0.0782	0.0908	0.0925	0.1210	0.0093	0.0096	0.0167	0.0135	0.0188	0.0170	0.0138	0.0230	0.0320	0.0380
Veneto	0.3779	0.3154	0.2565	0.2413	0.1989	0.1676	0.1554	0.1563	0.1473	0.1284	0.0272	0.0282	0.0401	0.0384	0.0305	0.0563	0.0541	0.0552	0.0621	0.0606
Friuli-Venezia Giulia	0.3363	0.2896	0.2290	0.2001	0.1456	0.1935	0.1875	0.1672	0.1532	0.1192	0.0371	0.0452	0.0481	0.0671	0.0541	0.0697	0.1206	0.1563	0.1409	0.0632
Liguria	0.0295	0.0218	0.0822	0.0350	0.0625	0.0683	0.1146	0.1166	0.1389	0.1940	0.0139	0.0163	0.0256	0.0226	0.0429	0.0283	0.0482	0.0731	0.1040	0.1354
Emilia-Romagna	0.3413	0.2671	0.2573	0.2121	0.1980	0.1764	0.1552	0.1258	0.1270	0.1325	0.0330	0.0244	0.0375	0.0278	0.0624	0.0804	0.0759	0.0697	0.0667	0.0759
Tuscany	0.3828	0.2896	0.2533	0.2063	0.2233	0.1735	0.1681	0.1316	0.1174	0.1200	0.0374	0.0211	0.0484	0.0394	0.0716	0.0551	0.0629	0.0482	0.0415	0.0541
Umbria	0.3032	0.3006	0.2437	0.3032	0.2221	0.2300	0.1952	0.1761	0.1413	0.1238	0.0447	0.0564	0.0540	0.0897	0.0854	0.1099	0.1061	0.1276	0.1172	0.1053
Marche	0.3640	0.3231	0.2304	0.2375	0.2453	0.1803	0.1776	0.2043	0.1782	0.2554	0.0935	0.0541	0.0482	0.0717	0.0858	0.1192	0.1533	0.1772	0.1898	0.2141
Lazio	0.3480	0.3504	0.3270	0.3170	0.2958	0.2596	0.2514	0.2625	0.2829	0.3087	0.0185	0.0251	0.0545	0.1032	0.1630	0.2073	0.2356	0.2777	0.3081	0.3374
Abruzzo	0.3990	0.4891	0.3895	0.3694	0.3728	0.3858	0.3536	0.2997	0.2916	0.3074	0.0574	0.0792	0.0750	0.1266	0.1923	0.2855	0.3513	0.3516	0.3150	0.3216
Molise	0.3765	0.4093	0.3011	0.2993	0.3100	0.3318	0.3286	0.3299	0.2861	0.2406	0.0662	0.0776	0.1340	0.1578	0.2457	0.3414	0.3582	0.3837	0.3117	0.3296
Campania	0.0091	0.0313	0.0703	0.1466	0.2400	0.2838	0.3282	0.3776	0.4059	0.3839	0.0031	0.0090	0.0418	0.0582	0.1781	0.3269	0.4321	0.5275	0.5391	0.5223
Apulia	0.3408	0.2829	0.2732	0.2332	0.2304	0.2515	0.2319	0.2300	0.2064	0.2396	0.0192	0.0186	0.0433	0.0995	0.0965	0.1667	0.2489	0.2566	0.2562	0.2699
Basilicata	0.2608	0.3732	0.3262	0.2737	0.3294	0.2734	0.3244	0.3015	0.3056	0.2674	0.0469	0.0606	0.1454	0.2119	0.1304	0.2395	0.3046	0.4339	0.3437	0.3200
Calabria	0.3351	0.3077	0.3617	0.3333	0.3379	0.3508	0.3014	0.2787	0.2696	0.2658	0.0252	0.0457	0.0546	0.1122	0.1352	0.2618	0.3584	0.3719	0.3598	0.3547
Sicily	0.2966	0.2965	0.2746	0.2706	0.2013	0.2549	0.2177	0.2152	0.1802	0.1857	0.0077	0.0191	0.0397	0.0619	0.1539	0.1770	0.1889	0.2182	0.1949	0.2115
Sardinia	0.5517	0.3420	0.2205	0.2291	0.2203	0.1611	0.1142	0.0725	0.0470	0.0536	0.0414	0.0330	0.0316	0.0606	0.0338	0.0298	0.0417	0.0262	0.0098	0.0079
Bolzano	0.5607	0.5217	0.4613	0.3766	0.3194	0.2194	0.2331	0.1573	0.1354	0.2213	0.1108	0.0836	0.0916	0.1001	0.1050	0.1433	0.1271	0.1471	0.1407	0.0808
Trento	0.5138	0.4323	0.2887	0.2406	0.1796	0.1744	0.0847	0.0892	0.0605	0.1012	0.1033	0.0453	0.0881	0.0510	0.0603	0.0778	0.0417	0.0355	0.0399	0.0184
(e) ${\hat{\lambda }}_{i,5}^{\left(1\right)}$																				
Piedmont	0.1311	0.2530	0.2568	0.3332	0.3715	0.3146	0.2959	0.2664	0.1741	0.1136	0.0972	0.1082	0.2215	0.1205	0.2086	0.2733	0.2495	0.2337	0.2347	0.1688
Aosta Valley	0.0960	0.1146	0.2494	0.1752	0.2103	0.3083	0.1597	0.1022	0.1653	0.1520	0.1459	0.1968	0.1468	0.1428	0.1520	0.1873	0.1397	0.2151	0.1335	0.1025
Lombardy	0.3268	0.5206	0.6397	0.7222	0.6937	0.6661	0.5534	0.4270	0.2946	0.1565	0.2078	0.2384	0.2701	0.3549	0.4463	0.5078	0.4943	0.4982	0.5259	0.4702
Veneto	0.0578	0.1078	0.0589	0.0654	0.0469	0.0729	0.0475	0.0406	0.0348	0.0269	0.0663	0.0417	0.0531	0.0332	0.0346	0.0382	0.0467	0.0607	0.0999	0.0819
Friuli-Venezia Giulia	0.0974	0.1098	0.1101	0.0946	0.0740	0.1188	0.0900	0.0661	0.0751	0.0802	0.0670	0.0934	0.1083	0.0513	0.0568	0.1203	0.0635	0.0980	0.1210	0.1140
Liguria	0.0538	0.0822	0.1784	0.1919	0.2615	0.2856	0.2218	0.2030	0.1383	0.1483	0.0714	0.0926	0.1036	0.1838	0.1781	0.2481	0.1973	0.1697	0.1807	0.1478
Emilia-Romagna	0.1317	0.2215	0.2977	0.2845	0.3117	0.3026	0.2437	0.1901	0.1148	0.0631	0.0676	0.1023	0.1331	0.2060	0.1890	0.2514	0.1932	0.1886	0.1799	0.0879
Tuscany	0.0594	0.1895	0.1412	0.1893	0.1184	0.1094	0.1099	0.0880	0.0649	0.0504	0.1028	0.1020	0.0573	0.0725	0.0990	0.0931	0.0730	0.0423	0.0437	0.0359
Umbria	0.1733	0.1968	0.1117	0.1326	0.0853	0.0750	0.0456	0.0615	0.0878	0.0910	0.1290	0.1348	0.0981	0.1611	0.0816	0.0912	0.0701	0.0538	0.0678	0.0777
Marche	0.0831	0.1949	0.1618	0.2600	0.2177	0.1914	0.2148	0.1479	0.0979	0.0805	0.0981	0.0860	0.1467	0.1131	0.0742	0.1240	0.1089	0.0905	0.0878	0.0645
Lazio	0.1919	0.1048	0.1430	0.0943	0.0566	0.0752	0.0627	0.0346	0.0391	0.0204	0.0780	0.0868	0.0734	0.0766	0.0687	0.0649	0.0611	0.0452	0.0439	0.0231
Abruzzo	0.1257	0.2084	0.2535	0.1578	0.1366	0.1256	0.0771	0.0528	0.0615	0.0535	0.1162	0.1827	0.1420	0.1164	0.1251	0.1064	0.0742	0.0814	0.0984	0.0465
Molise	0.1325	0.1414	0.1213	0.1464	0.1190	0.1126	0.0911	0.0711	0.0938	0.1116	0.2045	0.1482	0.1956	0.1230	0.1204	0.0944	0.0829	0.0866	0.0856	0.0532
Campania	0.0620	0.1233	0.0772	0.1022	0.0654	0.0609	0.0238	0.0158	0.0124	0.0180	0.0202	0.0459	0.0959	0.0750	0.0681	0.0687	0.0295	0.0445	0.0452	0.0310
Apulia	0.1292	0.1855	0.1187	0.0899	0.0568	0.0740	0.0671	0.0336	0.0652	0.0448	0.0838	0.1119	0.0749	0.1011	0.0868	0.0752	0.0648	0.0815	0.0377	0.0329
Basilicata	0.1458	0.1023	0.1134	0.1138	0.1805	0.0912	0.0738	0.0591	0.0644	0.0868	0.1330	0.0910	0.1812	0.1519	0.1127	0.0840	0.0963	0.0835	0.1176	0.1232
Calabria	0.0935	0.1185	0.1042	0.1338	0.0810	0.0709	0.0356	0.0273	0.0430	0.0438	0.0832	0.1009	0.1020	0.0957	0.0638	0.0615	0.0596	0.0578	0.0331	0.0318
Sicily	0.0898	0.0997	0.0824	0.0946	0.0800	0.0434	0.0225	0.0152	0.0172	0.0255	0.0717	0.0901	0.1213	0.1157	0.0619	0.0669	0.0342	0.0195	0.0357	0.0286
Sardinia	0.0877	0.1196	0.1768	0.0950	0.0731	0.0584	0.0435	0.0641	0.0418	0.0473	0.0907	0.0633	0.0947	0.0708	0.0902	0.0642	0.0487	0.0726	0.0400	0.0373
Bolzano	0.0872	0.1067	0.1314	0.1039	0.1968	0.2081	0.1892	0.2130	0.1454	0.1266	0.1531	0.1394	0.1575	0.1563	0.0933	0.1715	0.1763	0.2409	0.1897	0.2353
Trento	0.0749	0.0782	0.0960	0.1138	0.1203	0.1482	0.0776	0.1383	0.1105	0.1309	0.1116	0.0854	0.0931	0.1184	0.1024	0.1324	0.1022	0.1547	0.1036	0.1472
(f) ${\hat{\lambda }}_{i,6}^{\left(1\right)}$																				
Piedmont	0.0678	0.0889	0.0979	0.1430	0.1657	0.2039	0.2426	0.3120	0.4211	0.4941	0.5065	0.3893	0.3005	0.1815	0.1281	0.0518	0.0421	0.0142	0.0147	0.0217
Aosta Valley	0.0496	0.0646	0.1495	0.0979	0.1680	0.1942	0.1943	0.2789	0.4678	0.2977	0.1994	0.1844	0.2606	0.2209	0.1952	0.1124	0.0632	0.0381	0.0332	0.0763
Lombardy	0.0529	0.0374	0.0969	0.1163	0.1603	0.2093	0.2708	0.3730	0.4790	0.6461	0.4626	0.3926	0.3005	0.2239	0.1554	0.1129	0.0735	0.0505	0.0526	0.0848
Veneto	0.0332	0.0498	0.0953	0.1477	0.1700	0.1947	0.2480	0.3140	0.4038	0.5086	0.4466	0.3217	0.2775	0.2015	0.1422	0.1097	0.0725	0.0330	0.0166	0.0393
Friuli-Venezia Giulia	0.0847	0.0643	0.0823	0.1874	0.2108	0.2415	0.2916	0.3695	0.4175	0.5118	0.5002	0.3970	0.2982	0.2667	0.2028	0.1859	0.1523	0.1205	0.1046	0.0918
Liguria	0.0315	0.0410	0.0549	0.0985	0.1483	0.1993	0.2462	0.3097	0.4146	0.5088	0.4003	0.3611	0.3050	0.1860	0.1315	0.0373	0.0191	0.0190	0.0321	0.0341
Emilia-Romagna	0.0501	0.0543	0.0927	0.1262	0.1609	0.1812	0.2407	0.3295	0.4432	0.5633	0.4633	0.3516	0.2654	0.1970	0.1607	0.1064	0.0806	0.0659	0.0689	0.1024
Tuscany	0.0390	0.0493	0.1238	0.1758	0.2120	0.2416	0.2873	0.3647	0.4382	0.4945	0.5276	0.3833	0.2849	0.2262	0.1485	0.0981	0.0739	0.0661	0.0717	0.0856
Umbria	0.0564	0.1008	0.1385	0.1368	0.1854	0.1920	0.2639	0.3620	0.3943	0.5217	0.3925	0.3504	0.2497	0.2226	0.1590	0.1268	0.0737	0.0521	0.0544	0.0715
Marche	0.0738	0.0624	0.1198	0.1407	0.1522	0.1682	0.2194	0.2829	0.3637	0.4384	0.3767	0.3329	0.2302	0.2404	0.1337	0.0898	0.0440	0.0178	0.0243	0.0261
Lazio	0.0185	0.0388	0.1278	0.1783	0.2378	0.2649	0.3178	0.3906	0.4403	0.4977	0.5989	0.4095	0.3113	0.2687	0.1797	0.1458	0.1145	0.1041	0.1086	0.1597
Abruzzo	0.0648	0.0410	0.0615	0.0907	0.1458	0.1983	0.2245	0.2886	0.3287	0.3698	0.3840	0.3123	0.1636	0.1769	0.0693	0.0453	0.0132	0.0082	0.0157	0.0242
Molise	0.0895	0.0751	0.1470	0.0838	0.1024	0.1634	0.2202	0.2058	0.2718	0.1597	0.2215	0.3054	0.1844	0.0799	0.0728	0.0371	0.0381	0.0327	0.0413	0.0475
Campania	0.0153	0.0784	0.1552	0.1836	0.2480	0.2724	0.3174	0.3429	0.3528	0.3137	0.4698	0.4624	0.2846	0.2192	0.1144	0.0667	0.0519	0.0428	0.0351	0.0445
Apulia	0.0237	0.0532	0.1488	0.1566	0.1905	0.2501	0.2841	0.3546	0.4041	0.4525	0.4294	0.3503	0.2006	0.1486	0.0572	0.0356	0.0285	0.0516	0.0516	0.0948
Basilicata	0.0520	0.0456	0.0882	0.1105	0.1162	0.2022	0.2245	0.2738	0.3221	0.2888	0.3218	0.3177	0.1404	0.1258	0.0751	0.0291	0.0247	0.0398	0.0413	0.0691
Calabria	0.0195	0.0293	0.0644	0.0940	0.1537	0.1561	0.2250	0.2585	0.2649	0.2745	0.4172	0.2839	0.1368	0.0731	0.0267	0.0182	0.0227	0.0102	0.0139	0.0146
Sicily	0.0113	0.0553	0.0947	0.1402	0.1724	0.2132	0.2616	0.2969	0.3176	0.3113	0.4758	0.3929	0.2049	0.1002	0.0199	0.0239	0.0115	0.0052	0.0081	0.0107
Sardinia	0.0190	0.0482	0.1109	0.1677	0.1921	0.2078	0.2446	0.2941	0.3326	0.3662	0.5244	0.3642	0.2699	0.1750	0.0946	0.0579	0.0340	0.0135	0.0176	0.0244
Bolzano	0.0317	0.0678	0.0919	0.1078	0.1400	0.1998	0.1986	0.2206	0.2832	0.3775	0.2846	0.2579	0.2524	0.1796	0.2049	0.0811	0.0754	0.0482	0.0268	0.0680
Trento	0.0330	0.0417	0.0939	0.1596	0.1892	0.2102	0.2467	0.2710	0.3613	0.4489	0.2959	0.2875	0.1882	0.1989	0.1634	0.1600	0.0989	0.0556	0.0486	0.0617

To further unpack these insights, a new matrix of dimension $21\times (2\times 10\times H_{1})$ is constructed by rearranging the posterior mean estimates of $\lambda_{i,h_{1}}^{\left(1\right)},i=1,\ldots ,N,h_{1}=1,\ldots ,H_{1}$. Here, 21 is the number of Italian regions, while 2 and 10 represent sex and age groups. We then classify Italian regions based on these features using the partitioning around medoids (PAM) algorithm. The optimal number of clusters, according to the elbow method, is 4.

**Figure 2.**
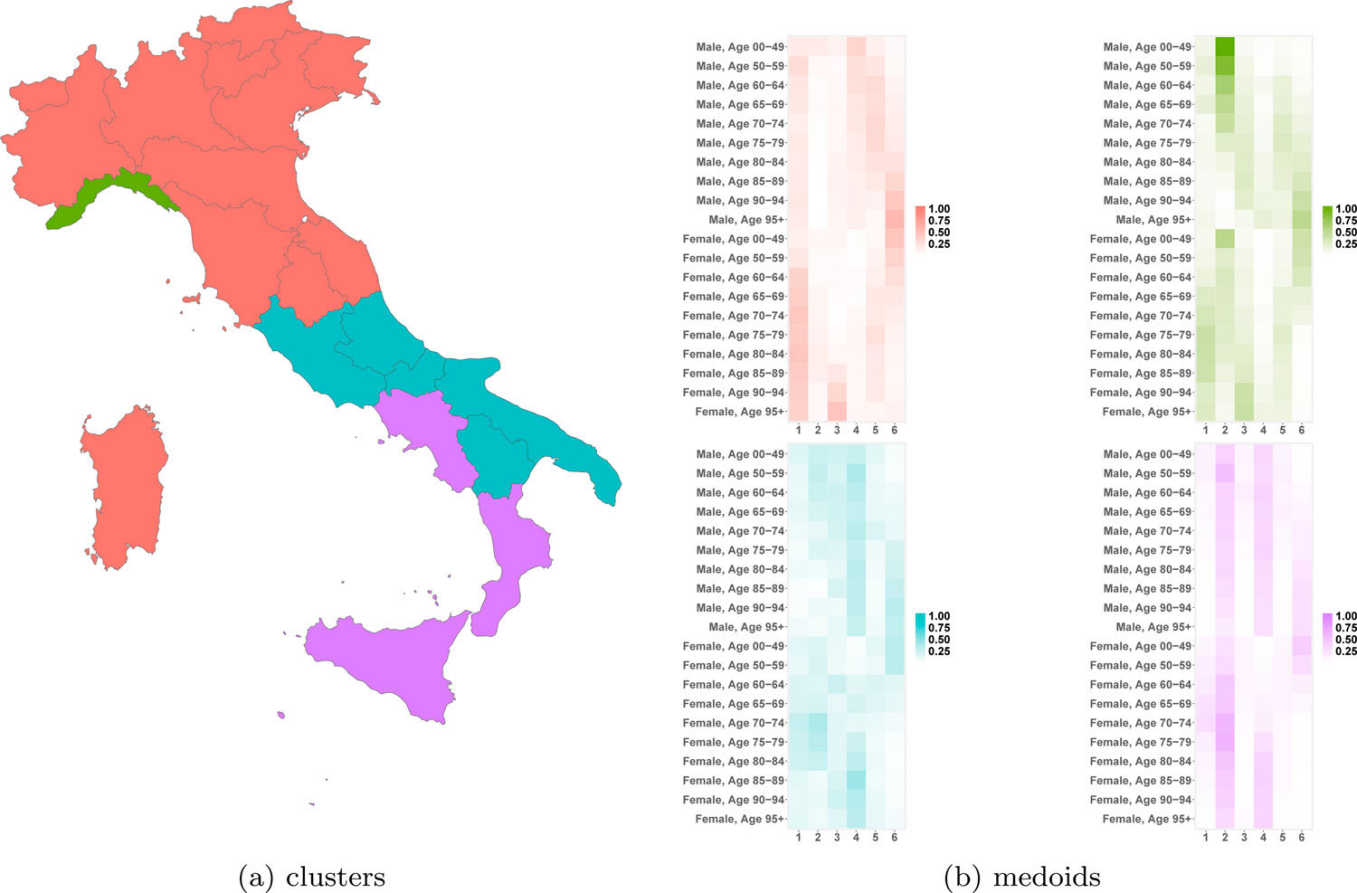
PAM classification of Italian regions based on $\lambda_{i,h_{1}}^{\left(1\right)}$. Horizontal axes in (b) denote latent classes. (a) clusters and (b) medoids.

The clustering algorithm corroborates previous observations. As displayed in Figure 2(a), northern Italy, along with Tuscany, Umbria, and Marche, is classified differently from southern Italy, plus Lazio and excluding Campania, Calabria, and Sicily. These two clusters have similar weights in latent class $h_{1}=6$, but exhibit differences in classes $h_{1}=1$ and $h_{1}=4$, notably for female populations in northern Italy and older females in southern Italy. Southern Italy is further divided into two groups that show homogeneous behavior in latent classes $h_{1}=4$ and $h_{1}=6$, but substantial differences in latent class $h_{2}$, as shown in Figure 2(b). Lastly, Liguria forms its own cluster due to the significant role of latent class $h_{1}=2$ in defining the region's mortality pattern over time.

**Figure 3.**
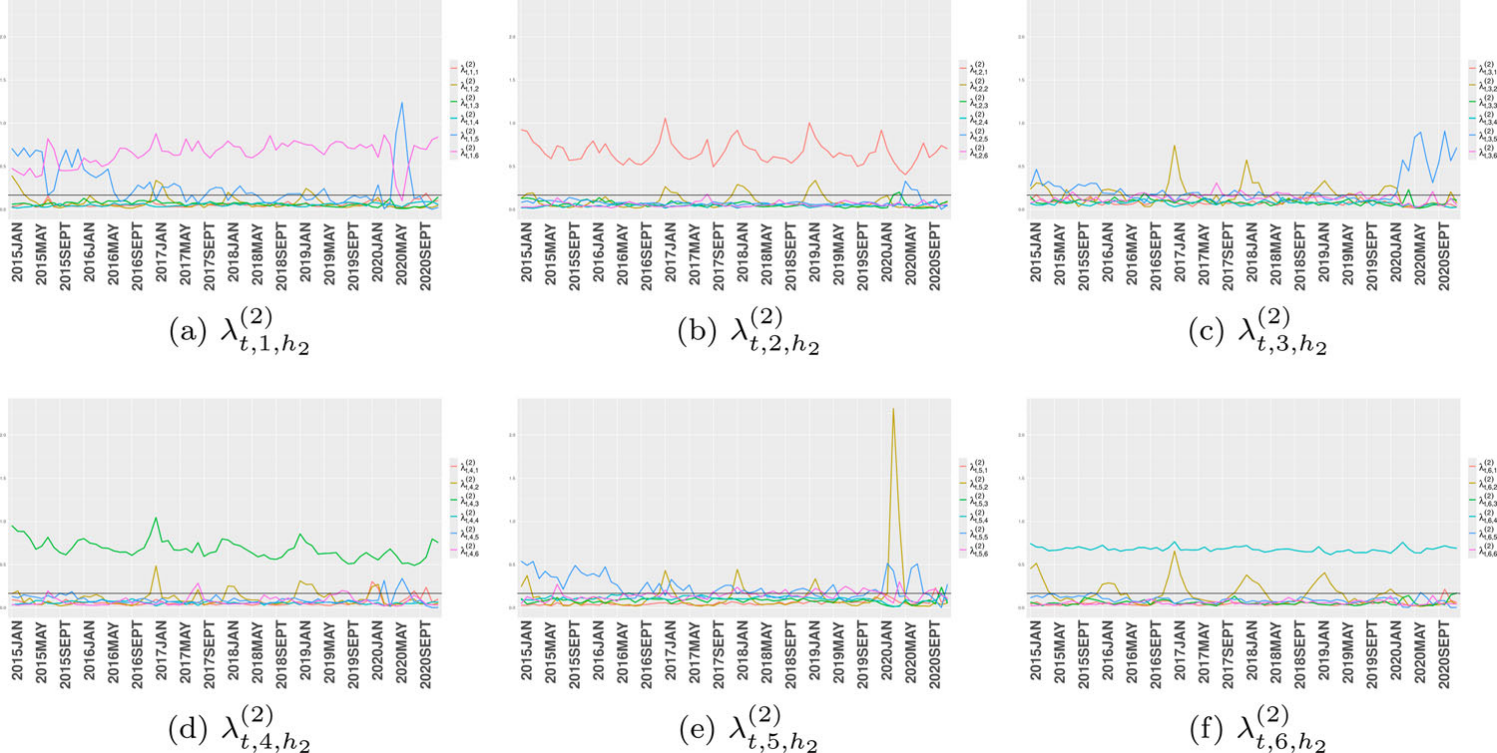
Trajectories of $\lambda_{t,h_{1},h_{2}}^{\left(2\right)}$ for each latent class $h_{1}=1,\ldots ,H_{1}$ from January 2015 to December 2020. Black horizontal lines represent the gamma prior mean $\gamma_{a}/\gamma_{b}$ for $\lambda_{t,h_{1},h_{2}}^{\left(2\right)}$. (a) $\lambda_{t,1,h_{2}}^{\left(2\right)}$. (b) $\lambda_{t,2,h_{2}}^{\left(2\right)}$. (c) $\lambda_{t,3,h_{2}}^{\left(2\right)}$. (d) $\lambda_{t,4,h_{2}}^{\left(2\right)}$. (e) $\lambda_{t,5,h_{2}}^{\left(2\right)}$ and (f) $\lambda_{t,6,h_{2}}^{\left(2\right)}$.

**Figure 4.**
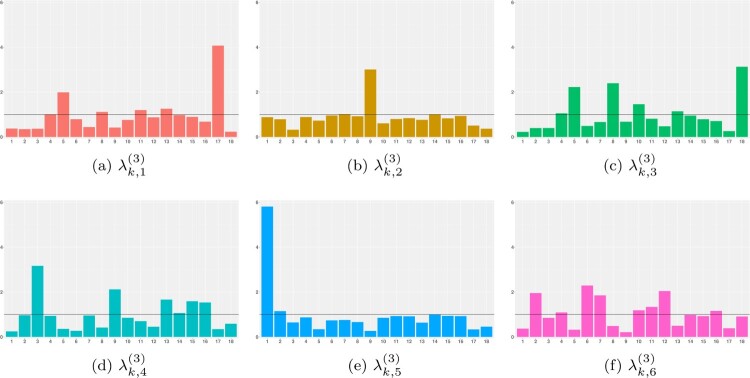
Bar plots of $\lambda_{k,h_{2}}^{\left(3\right)}$ for 18 causes of death (horizontal axes) for each latent class $h_{2}=1,\ldots ,H_{2}$. Black horizontal lines represent the gamma prior mean $\epsilon_{a}/\epsilon_{b}$ for $\lambda_{k,h_{2}}^{\left(3\right)}$. (a) $\lambda_{k,1}^{\left(3\right)}$. (b) $\lambda_{k,2}^{\left(3\right)}$. (c) $\lambda_{k,3}^{\left(3\right)}$. (d) $\lambda_{k,4}^{\left(3\right)}$. (e) $\lambda_{k,5}^{\left(3\right)}$ and (f) $\lambda_{k,6}^{\left(3\right)}$.

The second layer tensor cores $\lambda_{t,h_{1},h_{2}}^{\left(2\right)}$ and the third layer $\lambda_{k,h_{2}}^{\left(3\right)}$ jointly identify the corresponding latent classes denoted by $h_{1}$. The $\lambda_{t,h_{1},h_{2}}^{\left(2\right)}$ parameters correspond to time indices *T*, and their posterior mean estimates are shown as time-evolving trajectories in Figure 3. Meanwhile, $\lambda_{k,h_{2}}^{\left(3\right)}$ assumes $H_{2}$ latent structures that summarize 18 causes of death, as displayed in Figure 4.

We begin our analysis with latent class $h_{1}=1$, relevant to most female age groups barring the older population in southern Italy. Two trajectories within this class, characterized by mortality rates $\lambda_{k,5}^{\left(3\right)}$ and $\lambda_{k,6}^{\left(3\right)}$, are particularly significant. $\lambda_{k,5}^{\left(3\right)}$ primarily captures COVID-19 mortality. Its corresponding trajectory $\lambda_{t,1,5}^{\left(2\right)}$ showcases a sudden weight spike for latent class $h_{2}=5$ around June 2020, a period of relative calm between the first and second waves of the pandemic. This could be attributed to the time lag between COVID-19 infection during the previous wave and subsequent deaths. Another spike related to this latent class will be discussed later. The trajectory $\lambda_{t,1,6}^{\left(2\right)}$ demonstrates a counter-behavior to $\lambda_{t,1,5}^{\left(2\right)}$; it decreases when the latter rises and vice versa. Notable causes of death represented by this trajectory ( $\lambda_{k,6}^{\left(3\right)}$) include infectious and parasitic diseases; psychic and behavioral disorders; diseases of the nervous system and sense organs; digestive system diseases; diseases of the skin and subcutaneous tissue; and diseases of the musculoskeletal system and connective tissue. Although the Poisson regression component revealed a global positive main effect of COVID lockdown measures on the mortality rate of psychic and behavioral disorders, this countervailing phenomenon does not contradict earlier arguments. As the discussed latent class $h_{1}=1$ is crucial to the female population, barring older ones in southern Italy, it instead suggests a local compensation effect specific to this demographic group.

Latent class $h_{1}=2$ is specific to the southern Italian regions of Campania, Calabria, and Sicily. The key trajectory $\lambda_{t,2,1}^{\left(2\right)}$ within this class, shown in Figure 3(b), exhibits high estimated mortality rates for endocrine, nutritional, and metabolic diseases as well as symptoms, signs, abnormal results, and ill-defined causes (Figure 4(a)). This trajectory shows strong seasonality with peaks in both winter and summer. Previous research suggests links between winter holidays and heat exposure with endocrine, nutritional, and metabolic diseases [32,44]. The seasonal pattern of symptoms, signs, abnormal results, and ill-defined causes may involve misclassified deaths related to seasonal illnesses.

Latent class $h_{1}=3$ primarily explains mortality in females older than 85 years in northern Italy and certain male age groups in the south. This class depicts a pattern of COVID-19 mortality with two spikes in June 2020 and October 2020 (Figure 4(c)). The spike at the end of the first wave may result from the time lag between contracting and dying from COVID-19, as commented when analyzing latent class $h_{1}=1$. The October spike anticipates the second COVID-19 wave, potentially due to factors such as insufficient testing and reporting, as well as unprepared health systems. We identify two types of displacement between case peak and mortality peak. The first type arises from the time lag between infection and death from COVID-19, while the second type predicts incoming COVID-19 waves. The latter displacement was particularly true in 2020 when societal and health system preparedness for the pandemic was low.

Latent class $h_{1}=4$ represents the mortality composition of young male Italians in the north and all populations in the south. The defining feature of this class is the downward trend of trajectory $\lambda_{t,4,3}^{\left(2\right)}$ (Figure 3(d)). Further inspection of Figure 4(c) reveals that endocrine, nutritional, and metabolic diseases; diseases of the circulatory system; and external causes of trauma and poisoning define the mortality structure in $\lambda_{k,3}^{\left(3\right)}$. These mortality causes tend to be seasonal, with different effects in northern Italy and the south, barring Campania, Calabria, and Sicily. For instance, a 2017 heatwave caused a noticeable increase in deaths from endocrine, nutritional, and metabolic diseases in Campania, Calabria, and Sicily, but the effect was less pronounced in the north. Additionally, these diseases are more lethal for the older female population, as indicated in Table 3(b) and Table 3(d). Seasonality in deaths from diseases of the circulatory system aligns with prior research [12,39]. Lastly, the seasonality of external causes of trauma and poisoning may largely result from increased traffic accidents in the winter and outdoor activities in the summer.

Latent class $h_{1}=5$, which is primarily significant for both males and females in northern Italy, can be characterized by two main features. Firstly, the trajectory $\lambda_{t,5,2}^{\left(2\right)}$, which represents the mortality rate of diseases of the respiratory system, displays a notable spike around March and April 2020. This is a time when the health system in northern Italy was overwhelmed and many COVID-19 related deaths may have been misclassified. A similar point has been raised when interpreting coefficients of the Poisson regression component. The second key feature of this latent class is the trajectory $\lambda_{t,5,5}^{\left(2\right)}$, which peaks twice: first in February and then again in July 2020. Both types of displacement of COVID-19 mortality rate appear in this class. The second type of displacement, which precedes the first wave of COVID-19 (February and March 2020), is experienced by almost all males aged between 50 and 89 and females aged between 70 and 94 in northern Italy, with the exceptions of Veneto and Friuli-Venezia Giulia. In contrast, the first type of displacement occurs only for the older female population in northern Italy and certain male age groups in the south at the beginning of the second wave, as previously illustrated.

Lastly, latent class $h_{1}=6$ in Figure 3(f) is characterized by a constant trend of $\lambda_{t,6,4}^{\left(2\right)}$, which is primarily defined by tumor and respiratory diseases (Figure 4(d)). Another notable trajectory within this class is $\lambda_{t,6,2}^{\left(2\right)}$, which captures the expected seasonality of respiratory disease deaths. This mortality structure is common to older male populations and females under 69 across nearly all Italian regions.

## 6. Summary and future work

In this paper, we propose to model Poisson count data using the BPRTTD model. The model comprises two components: a Poisson regression model and a tensor train decomposition applied to the data organized as a tensor for estimating the latent parameter space. The model and the Bayesian approach are validated via two simulation studies and applied to monthly Italian mortality data, segmented by cause, from January 2015 to December 2020. The regression component of our model effectively leverages covariate information, allowing us to identify causes of death positively, negatively, and non-related to government interventions during the COVID-19 pandemic. The tensor decomposition component enables further stratification of demographic profiles, based on unique dynamic mortality structures over time. This is achieved by jointly characterizing profiles by geographical location, sex, and age. Regional classifications are made, and the results align with conventional conceptions. The impact of COVID-19 is also revealed in latent tensor cores, with several causes of death, including infectious and parasitic diseases and psychic and behavioral disorders, competing with COVID-19 mortality among specific demographic groups. In the application, we arrange the data into a three-way tensor, but the proposed methods can be directly applied to tensors of higher orders. The posterior sampling algorithm needs to be adjusted accordingly, but no major conceptual changes are required. However, we have not fully exploited the spatial-temporal information in the data. For instance, instead of applying clustering algorithms to the posterior estimates, one can introduce a reasonable metric and utilize geographic locations encoded in $\lambda_{i,h_{1}}^{\left(1\right)}$ when specifying the model. $\lambda_{t,h_{1},h_{2}}^{\left(2\right)}$ can also be modeled in a time series framework so that temporal dependence can be inferred. Another potential direction for future research involves the selection of tensor train ranks in the BPRTTD model, which plays a critical role in controlling model complexity. Model selection could be achieved by calculating marginal likelihoods over pre-specified grids defined by tensor train ranks. However, given the substantial computational load this would require, we reserve this exploration for future work.

## Supplementary Material

BPRTTD_JAS_supplementary material.pdf
