# Supplementary material for: Bayesian poisson regression tensor train decomposition model for learning mortality pattern changes during COVID-19 pandemic
Source: J Appl Stat. 2024 Oct 10;52(5):1017–39. doi: 10.1080/02664763.2024.2411608 (PMC11951336; doi:10.1080/02664763.2024.2411608)
Supplement: BPRTTD_JAS_supplementary material.pdf [file CJAS_A_2411608_SM0501.pdf]

Supplementary Material for Bayesian Poisson Regression Tensor Train Decomposition Model for Learning Mortality Pattern Changes during COVID-19 Pandemic

Wei Zhang[a], Antonietta Mira [b] and Ernst C. Wit [a]

[a]Faculty of Informatics, Università della Svizzera italiana, 6900, Lugano, Switzerland
[b]Faculty of Economics, Euler Institute, Università della Svizzera italiana, 6900, Lugano, Switzerland and Insubria University

ARTICLE HISTORY
Compiled July 30, 2024

1. Simulation study where parameters are artificially generated

In this section, we show how the true values of the parameters $\lambda^{(1)}_{i,h_1}, \lambda^{(2)}_{t,h_1,h_2}, \lambda^{(3)}_{k,h_2}$ are recovered using our method. For $\lambda^{(1)}_{i,h_1}$, the difference between the 100 true values and their estimates by posterior means has an average of 0.0148. The difference is 0.0562 and -0.0675 for $\lambda^{(2)}_{t,h_1,h_2}$ and $\lambda^{(3)}_{k,h_2}$ respectively, validating our approach's ability to recover the true values of the parameters. Since BPRCPD model estimates a completely different set of parameters, we are not able to directly compare the performance in terms of the difference between true parameters and the estimated ones as in Tables 1 - 3. We instead calculate the deviance statistic commonly used in Poisson regression to evaluate the goodness-of-fit. The results are displayed in Table 4. It is easy to observe that, as expected, the higher is the number of parameters in the model, the smaller is the deviance statistic, reflecting a better fit to the data.

CONTACT Wei Zhang. Email: wei.zhang@usi.ch

Table 1. Comparison between true $\lambda^{(1)}_{i,h_1}, i = 1, \ldots, N, h_1 = 1, \ldots, H_1$ and the estimated values from the BPRTTD model in terms of posterior mean. Average (*italic*) and standard deviation (*italic* in parentheses) of the posterior means over 100 repetitions are reported.

$\lambda^{(1)}_{i,h_1}$	$h_1 = 1$	$h_1 = 2$	$h_1 = 3$	$h_1 = 4$	$h_1 = 5$		$h_1 = 1$	$h_1 = 2$	$h_1 = 3$	$h_1 = 4$	$h_1 = 5$
$i = 1$	0.2499<br>*0.2594*<br>*(0.0940)*	0.7428<br>*0.8633*<br>*(0.1430)*	0.7504<br>*0.5378*<br>*(0.1631)*	0.1228<br>*0.2411*<br>*(0.1024)*	1.5231<br>*1.4893*<br>*(0.1421)*	$i = 11$	0.1982<br>*0.2051*<br>*(0.0470)*	0.0521<br>*0.0756*<br>*(0.0311)*	0.1117<br>*0.1505*<br>*(0.0534)*	0.5681<br>*0.5364*<br>*(0.0659)*	0.0095<br>*0.0548*<br>*(0.0243)*
$i = 2$	0.4828<br>*0.4953*<br>*(0.0561)*	0.0422<br>*0.0680*<br>*(0.0319)*	0.0125<br>*0.0621*<br>*(0.0278)*	0.1899<br>*0.1935*<br>*(0.0520)*	0.1153<br>*0.0956*<br>*(0.0329)*	$i = 12$	0.0247<br>*0.0400*<br>*(0.0189)*	0.1820<br>*0.1979*<br>*(0.0391)*	0.0872<br>*0.0743*<br>*(0.0329)*	0.2041<br>*0.1989*<br>*(0.0496)*	0.1542<br>*0.1630*<br>*(0.0407)*
$i = 3$	0.1814<br>*0.1656*<br>*(0.0569)*	0.3639<br>*0.4869*<br>*(0.0984)*	0.8121<br>*0.7108*<br>*(0.0998)*	0.1475<br>*0.1304*<br>*(0.0597)*	0.1404<br>*0.1893*<br>*(0.0622)*	$i = 13$	0.5626<br>*0.5979*<br>*(0.0913)*	0.1343<br>*0.1700*<br>*(0.0691)*	0.1817<br>*0.2817*<br>*(0.0944)*	1.1993<br>*1.1819*<br>*(0.1200)*	0.2786<br>*0.3102*<br>*(0.0868)*
$i = 4$	0.8794<br>*0.8870*<br>*(0.0910)*	0.3297<br>*0.3779*<br>*(0.0834)*	0.1031<br>*0.0987*<br>*(0.0496)*	0.0315<br>*0.0966*<br>*(0.0389)*	0.0903<br>*0.0802*<br>*(0.0343)*	$i = 14$	0.6064<br>*0.6226*<br>*(0.0834)*	0.0782<br>*0.1169*<br>*(0.0506)*	0.1485<br>*0.1830*<br>*(0.0625)*	0.3262<br>*0.3481*<br>*(0.0772)*	0.2202<br>*0.2066*<br>*(0.0606)*
$i = 5$	0.0098<br>*0.0493*<br>*(0.0264)*	0.0641<br>*0.0714*<br>*(0.0316)*	0.2238<br>*0.1997*<br>*(0.0681)*	0.3579<br>*0.3726*<br>*(0.0892)*	0.7434<br>*0.7269*<br>*(0.0804)*	$i = 15$	0.0655<br>*0.0743*<br>*(0.0293)*	0.2409<br>*0.2764*<br>*(0.0565)*	0.2397<br>*0.1706*<br>*(0.0630)*	0.0565<br>*0.0988*<br>*(0.0405)*	0.4995<br>*0.4861*<br>*(0.0606)*
$i = 6$	0.0565<br>*0.0645*<br>*(0.0251)*	0.1891<br>*0.2667*<br>*(0.0732)*	0.5013<br>*0.4558*<br>*(0.0728)*	0.3215<br>*0.2744*<br>*(0.0687)*	0.0514<br>*0.1042*<br>*(0.0376)*	$i = 16$	0.2084<br>*0.2028*<br>*(0.0601)*	0.0165<br>*0.1240*<br>*(0.0835)*	0.8185<br>*0.7796*<br>*(0.1080)*	0.3302<br>*0.2697*<br>*(0.0884)*	0.1027<br>*0.1522*<br>*(0.0546)*
$i = 7$	0.7599<br>*0.7897*<br>*(0.0834)*	0.0972<br>*0.1186*<br>*(0.0567)*	0.0843<br>*0.0994*<br>*(0.0441)*	0.1474<br>*0.2371*<br>*(0.0825)*	1.0067<br>*0.9216*<br>*(0.0904)*	$i = 17$	0.8964<br>*0.9180*<br>*(0.1053)*	0.2100<br>*0.2673*<br>*(0.0828)*	0.2178<br>*0.2105*<br>*(0.0650)*	0.0905<br>*0.1561*<br>*(0.0616)*	0.1804<br>*0.1505*<br>*(0.0525)*
$i = 8$	0.8106<br>*0.8290*<br>*(0.0915)*	0.2725<br>*0.2718*<br>*(0.0822)*	0.0027<br>*0.0388*<br>*(0.0152)*	0.0330<br>*0.0714*<br>*(0.0293)*	0.0225<br>*0.0324*<br>*(0.0109)*	$i = 18$	0.1017<br>*0.1084*<br>*(0.0373)*	0.0512<br>*0.0819*<br>*(0.0314)*	0.1591<br>*0.1787*<br>*(0.0508)*	0.4552<br>*0.4293*<br>*(0.0604)*	0.0883<br>*0.1166*<br>*(0.0343)*
$i = 9$	1.0723<br>*1.1206*<br>*(0.1554)*	0.0295<br>*0.1356*<br>*(0.0739)*	0.8724<br>*0.9154*<br>*(0.1932)*	1.4174<br>*1.4608*<br>*(0.2246)*	1.5566<br>*1.5256*<br>*(0.1836)*	$i = 19$	0.4859<br>*0.5073*<br>*(0.0663)*	0.1011<br>*0.1583*<br>*(0.0561)*	0.3093<br>*0.3363*<br>*(0.0805)*	0.5595<br>*0.5516*<br>*(0.0977)*	0.2867<br>*0.2929*<br>*(0.0632)*
$i = 10$	0.2946<br>*0.2999*<br>*(0.0528)*	0.1376<br>*0.2085*<br>*(0.0559)*	0.4403<br>*0.4157*<br>*(0.0595)*	0.2664<br>*0.2475*<br>*(0.0632)*	0.0798<br>*0.1136*<br>*(0.0453)*	$i = 20$	0.1186<br>*0.1430*<br>*(0.0474)*	0.0476<br>*0.0660*<br>*(0.0289)*	0.1438<br>*0.1379*<br>*(0.0551)*	0.3574<br>*0.3716*<br>*(0.0763)*	0.7527<br>*0.7311*<br>*(0.0827)*

Table 2. Comparison between true $\lambda^{(2)}_{t,h_1,h_2}, t = 1, \ldots, T, h_1 = 1, \ldots, H_1, h_2 = 1, \ldots, H_2$ and the estimated values from the BPRTTD model in terms of posterior mean. Average (*italic*) and standard deviation (*italic* in parentheses) of the posterior means over 100 repetitions are reported.

	$h_2 = 1$	$h_2 = 2$	$h_2 = 3$	$h_2 = 4$	$h_2 = 5$
$\lambda^{(2)}_{1,h_1,h_2}$					
$h_1 = 1$	0.0797<br>*0.1100*<br>*(0.0488)*	0.4105<br>*0.2480*<br>*(0.1117)*	0.1303<br>*0.2151*<br>*(0.1042)*	0.0677<br>*0.1790*<br>*(0.0826)*	0.7889<br>*0.7200*<br>*(0.1903)*
$h_1 = 2$	0.0023<br>*0.1902*<br>*(0.0887)*	0.1695<br>*0.2872*<br>*(0.1387)*	0.3604<br>*0.4397*<br>*(0.1702)*	0.2775<br>*0.3485*<br>*(0.1533)*	0.1865<br>*0.3652*<br>*(0.1360)*
$h_1 = 3$	0.5591<br>*0.5592*<br>*(0.1986)*	0.2067<br>*0.3226*<br>*(0.1156)*	0.6209<br>*0.6666*<br>*(0.2391)*	0.5517<br>*0.4520*<br>*(0.1814)*	0.1677<br>*0.5069*<br>*(0.2079)*
$h_1 = 4$	0.6189<br>*0.5389*<br>*(0.1622)*	0.2364<br>*0.2091*<br>*(0.0949)*	0.1666<br>*0.2376*<br>*(0.0979)*	0.1070<br>*0.2534*<br>*(0.1236)*	0.8827<br>*0.8649*<br>*(0.2575)*
$h_1 = 5$	0.4161<br>*0.5317*<br>*(0.1516)*	0.0383<br>*0.1738*<br>*(0.0554)*	0.2978<br>*0.2931*<br>*(0.1263)*	1.3384<br>*1.4089*<br>*(0.2299)*	0.2038<br>*0.3825*<br>*(0.1552)*
$\lambda^{(2)}_{2,h_1,h_2}$					
$h_1 = 1$	0.9319<br>*0.8287*<br>*(0.1669)*	0.2189<br>*0.2215*<br>*(0.0905)*	0.2541<br>*0.2365*<br>*(0.0980)*	0.4092<br>*0.4475*<br>*(0.1813)*	1.5906<br>*1.7110*<br>*(0.2320)*
$h_1 = 2$	0.0679<br>*0.2540*<br>*(0.1046)*	0.6289<br>*0.4702*<br>*(0.2187)*	0.1782<br>*0.4260*<br>*(0.1737)*	0.0160<br>*0.3503*<br>*(0.1762)*	0.1146<br>*0.3524*<br>*(0.1464)*
$h_1 = 3$	0.2926<br>*0.4065*<br>*(0.1530)*	0.9343<br>*1.0587*<br>*(0.2395)*	0.6891<br>*0.6539*<br>*(0.2308)*	0.4450<br>*0.3814*<br>*(0.1817)*	0.0428<br>*0.4280*<br>*(0.1927)*
$h_1 = 4$	0.7258<br>*0.7634*<br>*(0.1934)*	0.7789<br>*0.4645*<br>*(0.1746)*	0.0396<br>*0.2206*<br>*(0.0879)*	0.0306<br>*0.2670*<br>*(0.1089)*	0.7606<br>*0.6285*<br>*(0.2619)*
$h_1 = 5$	0.1564<br>*0.2273*<br>*(0.0949)*	0.1867<br>*0.2730*<br>*(0.1053)*	0.1724<br>*0.2580*<br>*(0.1170)*	0.7775<br>*0.7349*<br>*(0.1908)*	0.2383<br>*0.3370*<br>*(0.1550)*
$\lambda^{(2)}_{3,h_1,h_2}$					
$h_1 = 1$	0.0181<br>*0.0896*<br>*(0.0439)*	0.3822<br>*0.2729*<br>*(0.1047)*	0.1769<br>*0.2122*<br>*(0.0942)*	0.1921<br>*0.2436*<br>*(0.1109)*	0.0094<br>*0.1298*<br>*(0.0527)*
$h_1 = 2$	0.3461<br>*0.3476*<br>*(0.1335)*	0.3088<br>*0.3844*<br>*(0.1440)*	0.2935<br>*0.4390*<br>*(0.1901)*	0.9349<br>*0.7206*<br>*(0.2569)*	0.2371<br>*0.2926*<br>*(0.1151)*
$h_1 = 3$	0.3020<br>*0.2433*<br>*(0.0985)*	0.1293<br>*0.2531*<br>*(0.1148)*	0.4212<br>*0.3844*<br>*(0.1904)*	0.3518<br>*0.3858*<br>*(0.1956)*	0.0339<br>*0.2012*<br>*(0.0878)*
$h_1 = 4$	0.0460<br>*0.1312*<br>*(0.0644)*	0.4087<br>*0.2712*<br>*(0.1032)*	0.0540<br>*0.1961*<br>*(0.0680)*	0.4136<br>*0.3400*<br>*(0.1314)*	0.2308<br>*0.1906*<br>*(0.0783)*
$h_1 = 5$	0.0220<br>*0.1400*<br>*(0.0451)*	0.3266<br>*0.4058*<br>*(0.1595)*	0.6082<br>*0.6716*<br>*(0.1967)*	1.1064<br>*1.3000*<br>*(0.2372)*	0.0946<br>*0.2731*<br>*(0.1230)*
$\lambda^{(2)}_{4,h_1,h_2}$					
$h_1 = 1$	0.2536<br>*0.5279*<br>*(0.1546)*	2.3706<br>*2.0118*<br>*(0.2313)*	0.3284<br>*0.4373*<br>*(0.1935)*	2.1392<br>*2.1833*<br>*(0.3042)*	0.1644<br>*0.4292*<br>*(0.1736)*
$h_1 = 2$	0.0124<br>*0.2083*<br>*(0.0773)*	0.2341<br>*0.3964*<br>*(0.1663)*	0.4907<br>*0.6518*<br>*(0.2692)*	0.2338<br>*0.3236*<br>*(0.1202)*	0.2896<br>*0.3539*<br>*(0.1466)*
$h_1 = 3$	0.1771<br>*0.1786*<br>*(0.0690)*	0.3609<br>*0.4978*<br>*(0.2062)*	0.8364<br>*0.8339*<br>*(0.2657)*	0.0072<br>*0.2416*<br>*(0.0944)*	0.0286<br>*0.2418*<br>*(0.0889)*
$h_1 = 4$	0.1075<br>*0.2103*<br>*(0.0748)*	0.7630<br>*0.6093*<br>*(0.2106)*	0.3587<br>*0.4931*<br>*(0.2124)*	0.4609<br>*0.4042*<br>*(0.1701)*	0.2257<br>*0.3197*<br>*(0.1422)*
$h_1 = 5$	0.3143<br>*0.2306*<br>*(0.0956)*	0.0916<br>*0.3044*<br>*(0.1182)*	0.9410<br>*1.2383*<br>*(0.2491)*	0.3193<br>*0.3561*<br>*(0.1364)*	0.7090<br>*0.8282*<br>*(0.2515)*
$\lambda^{(2)}_{5,h_1,h_2}$					
$h_1 = 1$	0.4953<br>*0.5615*<br>*(0.1404)*	1.0245<br>*0.8780*<br>*(0.1635)*	0.2047<br>*0.2431*<br>*(0.0876)*	0.3998<br>*0.3610*<br>*(0.1206)*	0.1027<br>*0.2562*<br>*(0.1171)*
$h_1 = 2$	0.3414<br>*0.2743*<br>*(0.1105)*	0.0793<br>*0.2127*<br>*(0.0916)*	0.0311<br>*0.1845*<br>*(0.0628)*	0.0368<br>*0.2254*<br>*(0.0835)*	0.4831<br>*0.4198*<br>*(0.1981)*
$h_1 = 3$	0.1227<br>*0.2458*<br>*(0.1062)*	0.1466<br>*0.2352*<br>*(0.1013)*	0.1347<br>*0.1666*<br>*(0.0598)*	0.0710<br>*0.1958*<br>*(0.0862)*	0.5444<br>*0.3918*<br>*(0.1346)*
$h_1 = 4$	0.6107<br>*0.6006*<br>*(0.1626)*	0.9088<br>*0.7248*<br>*(0.2105)*	0.0928<br>*0.2121*<br>*(0.1049)*	0.2628<br>*0.2904*<br>*(0.1137)*	0.0626<br>*0.2197*<br>*(0.0799)*
$h_1 = 5$	0.1779<br>*0.2188*<br>*(0.0827)*	0.5038<br>*0.4956*<br>*(0.1574)*	0.5298<br>*0.6027*<br>*(0.1728)*	0.2453<br>*0.3244*<br>*(0.1281)*	0.7504<br>*0.9995*<br>*(0.2311)*
$\lambda^{(2)}_{6,h_1,h_2}$					
$h_1 = 1$	0.4075<br>*0.3896*<br>*(0.1158)*	0.1854<br>*0.1447*<br>*(0.0644)*	0.2374<br>*0.2152*<br>*(0.0919)*	0.0500<br>*0.2059*<br>*(0.0886)*	0.2034<br>*0.2658*<br>*(0.1131)*
$h_1 = 2$	0.9873<br>*0.7927*<br>*(0.2520)*	0.2300<br>*0.2353*<br>*(0.0872)*	0.1353<br>*0.4260*<br>*(0.1819)*	0.8357<br>*0.5288*<br>*(0.2267)*	0.3765<br>*0.3984*<br>*(0.1851)*
$h_1 = 3$	0.3378<br>*0.3453*<br>*(0.1623)*	0.0363<br>*0.2031*<br>*(0.0718)*	0.7779<br>*0.6603*<br>*(0.1910)*	0.0738<br>*0.3286*<br>*(0.1413)*	0.0102<br>*0.3205*<br>*(0.1375)*
$h_1 = 4$	0.5159<br>*0.5196*<br>*(0.1752)*	0.2163<br>*0.1625*<br>*(0.0699)*	0.1224<br>*0.2274*<br>*(0.0906)*	0.8139<br>*0.7292*<br>*(0.2386)*	1.0742<br>*1.1193*<br>*(0.2890)*
$h_1 = 5$	0.0286<br>*0.1678*<br>*(0.0831)*	0.0337<br>*0.0997*<br>*(0.0392)*	0.0916<br>*0.1765*<br>*(0.0635)*	0.7517<br>*0.7703*<br>*(0.2399)*	1.3187<br>*1.4628*<br>*(0.2842)*
$\lambda^{(2)}_{7,h_1,h_2}$					
$h_1 = 1$	0.1196<br>*0.2067*<br>*(0.0798)*	0.9589<br>*0.7249*<br>*(0.1723)*	0.2244<br>*0.3283*<br>*(0.1328)*	0.2025<br>*0.2990*<br>*(0.1142)*	0.2623<br>*0.3018*<br>*(0.1232)*
$h_1 = 2$	0.1234<br>*0.1990*<br>*(0.0899)*	0.9973<br>*0.9728*<br>*(0.3234)*	1.1448<br>*1.2515*<br>*(0.3687)*	0.2323<br>*0.3269*<br>*(0.1622)*	0.0813<br>*0.2650*<br>*(0.1168)*
$h_1 = 3$	0.0704<br>*0.2033*<br>*(0.0958)*	0.4072<br>*0.3376*<br>*(0.1307)*	0.5745<br>*0.4927*<br>*(0.2065)*	0.0742<br>*0.2971*<br>*(0.1191)*	0.0538<br>*0.2060*<br>*(0.0725)*
$h_1 = 4$	0.7759<br>*0.7611*<br>*(0.1492)*	0.0544<br>*0.1455*<br>*(0.0636)*	0.0351<br>*0.1555*<br>*(0.0555)*	1.0452<br>*0.8461*<br>*(0.2194)*	0.1444<br>*0.2092*<br>*(0.1028)*
$h_1 = 5$	0.3082<br>*0.3121*<br>*(0.1042)*	0.0481<br>*0.2005*<br>*(0.0850)*	0.2829<br>*0.3479*<br>*(0.1614)*	0.6157<br>*0.6495*<br>*(0.2040)*	0.0838<br>*0.1864*<br>*(0.0825)*
$\lambda^{(2)}_{8,h_1,h_2}$					
$h_1 = 1$	0.2924<br>*0.3252*<br>*(0.1190)*	0.5012<br>*0.3332*<br>*(0.1184)*	0.0018<br>*0.1806*<br>*(0.0696)*	0.4473<br>*0.4358*<br>*(0.1733)*	0.3434<br>*0.3225*<br>*(0.1316)*
$h_1 = 2$	0.1467<br>*0.3245*<br>*(0.1298)*	0.3038<br>*0.3953*<br>*(0.1726)*	0.5728<br>*0.5599*<br>*(0.3058)*	0.4417<br>*0.4305*<br>*(0.1754)*	0.1856<br>*0.3430*<br>*(0.1386)*
$h_1 = 3$	0.4748<br>*0.4069*<br>*(0.1373)*	0.1652<br>*0.3502*<br>*(0.1405)*	0.7937<br>*0.8440*<br>*(0.2612)*	0.2583<br>*0.4187*<br>*(0.1901)*	0.1159<br>*0.2905*<br>*(0.1104)*
$h_1 = 4$	0.2589<br>*0.3276*<br>*(0.1265)*	0.4934<br>*0.4842*<br>*(0.1797)*	0.5715<br>*0.5724*<br>*(0.2010)*	0.9355<br>*0.9110*<br>*(0.3072)*	0.1484<br>*0.3198*<br>*(0.1185)*
$h_1 = 5$	0.1349<br>*0.2727*<br>*(0.1001)*	0.5513<br>*0.5341*<br>*(0.1885)*	0.3935<br>*0.5538*<br>*(0.1937)*	1.2683<br>*1.4137*<br>*(0.2463)*	0.1405<br>*0.3073*<br>*(0.1212)*
$\lambda^{(2)}_{9,h_1,h_2}$					
$h_1 = 1$	0.5946<br>*0.5148*<br>*(0.1156)*	0.2287<br>*0.1773*<br>*(0.0774)*	0.2538<br>*0.2530*<br>*(0.1042)*	0.1797<br>*0.2467*<br>*(0.1072)*	0.2330<br>*0.2255*<br>*(0.0888)*
$h_1 = 2$	0.1888<br>*0.3641*<br>*(0.1381)*	0.1341<br>*0.4656*<br>*(0.2225)*	0.2881<br>*0.3197*<br>*(0.1165)*	0.5412<br>*0.4612*<br>*(0.1869)*	0.0934<br>*0.2599*<br>*(0.0946)*
$h_1 = 3$	0.1994<br>*0.3997*<br>*(0.1511)*	1.7503<br>*1.4674*<br>*(0.2576)*	0.1523<br>*0.3566*<br>*(0.1327)*	0.5286<br>*0.6506*<br>*(0.2363)*	0.0538<br>*0.2535*<br>*(0.1009)*
$h_1 = 4$	0.3296<br>*0.3521*<br>*(0.1357)*	0.4283<br>*0.4682*<br>*(0.1846)*	0.5576<br>*0.5541*<br>*(0.1854)*	0.8202<br>*0.7684*<br>*(0.1991)*	0.1152<br>*0.2813*<br>*(0.1205)*
$h_1 = 5$	0.1335<br>*0.1913*<br>*(0.0721)*	0.1763<br>*0.2447*<br>*(0.0946)*	0.1354<br>*0.2228*<br>*(0.0902)*	0.3890<br>*0.4420*<br>*(0.1560)*	0.2151<br>*0.2435*<br>*(0.0992)*
$\lambda^{(2)}_{10,h_1,h_2}$					
$h_1 = 1$	0.7580<br>*0.7046*<br>*(0.1253)*	0.2429<br>*0.2806*<br>*(0.1103)*	0.5086<br>*0.5552*<br>*(0.1753)*	0.5930<br>*0.5372*<br>*(0.1680)*	0.4208<br>*0.5201*<br>*(0.1947)*
$h_1 = 2$	0.3058<br>*0.3520*<br>*(0.1578)*	0.1734<br>*0.2102*<br>*(0.0725)*	0.0734<br>*0.3007*<br>*(0.1377)*	0.0286<br>*0.3231*<br>*(0.1302)*	0.4218<br>*0.3333*<br>*(0.1378)*
$h_1 = 3$	0.2769<br>*0.3211*<br>*(0.1513)*	0.2154<br>*0.3757*<br>*(0.1811)*	0.3063<br>*0.3765*<br>*(0.1335)*	0.7701<br>*0.6463*<br>*(0.2301)*	0.0960<br>*0.3698*<br>*(0.1644)*
$h_1 = 4$	0.0678<br>*0.1871*<br>*(0.0894)*	0.9899<br>*0.8184*<br>*(0.1839)*	0.3657<br>*0.5427*<br>*(0.2100)*	0.2727<br>*0.3576*<br>*(0.1522)*	0.6560<br>*0.6623*<br>*(0.2439)*
$h_1 = 5$	0.1034<br>*0.1222*<br>*(0.0468)*	0.2273<br>*0.2407*<br>*(0.0973)*	0.4600<br>*0.4899*<br>*(0.1695)*	0.0095<br>*0.1503*<br>*(0.0637)*	0.1234<br>*0.2326*<br>*(0.1208)*
$\lambda^{(2)}_{11,h_1,h_2}$					
$h_1 = 1$	0.2728<br>*0.2770*<br>*(0.0982)*	0.3415<br>*0.3144*<br>*(0.1235)*	0.2047<br>*0.3051*<br>*(0.1159)*	0.0946<br>*0.1850*<br>*(0.0831)*	0.6542<br>*0.5941*<br>*(0.1877)*
$h_1 = 2$	0.4223<br>*0.2812*<br>*(0.1102)*	1.0515<br>*0.7745*<br>*(0.2661)*	1.0073<br>*1.0988*<br>*(0.3340)*	0.0505<br>*0.2384*<br>*(0.0943)*	0.0372<br>*0.5056*<br>*(0.2088)*
$h_1 = 3$	0.0022<br>*0.1103*<br>*(0.0559)*	0.0388<br>*0.1695*<br>*(0.0702)*	0.3490<br>*0.3340*<br>*(0.1681)*	0.1090<br>*0.2034*<br>*(0.0783)*	0.5155<br>*0.3905*<br>*(0.1454)*
$h_1 = 4$	0.1444<br>*0.1652*<br>*(0.0740)*	0.2854<br>*0.1869*<br>*(0.0791)*	0.1641<br>*0.2257*<br>*(0.0893)*	0.4465<br>*0.3151*<br>*(0.1284)*	0.0134<br>*0.2676*<br>*(0.1345)*
$h_1 = 5$	0.1462<br>*0.1516*<br>*(0.0721)*	0.1240<br>*0.1661*<br>*(0.0677)*	0.1402<br>*0.2304*<br>*(0.0904)*	0.0866<br>*0.1944*<br>*(0.0875)*	0.7090<br>*0.7553*<br>*(0.1899)*
$\lambda^{(2)}_{12,h_1,h_2}$					
$h_1 = 1$	1.5351<br>*1.4388*<br>*(0.1669)*	0.0829<br>*0.2130*<br>*(0.0813)*	0.0924<br>*0.1327*<br>*(0.0492)*	0.0047<br>*0.1046*<br>*(0.0491)*	0.1651<br>*0.1214*<br>*(0.0461)*
$h_1 = 2$	0.7087<br>*0.6971*<br>*(0.2605)*	1.1825<br>*0.5893*<br>*(0.2295)*	0.1701<br>*0.2547*<br>*(0.1031)*	0.1212<br>*0.1757*<br>*(0.0589)*	0.0279<br>*0.1877*<br>*(0.0775)*
$h_1 = 3$	0.2001<br>*0.2553*<br>*(0.1109)*	0.2162<br>*0.3156*<br>*(0.1208)*	0.0974<br>*0.1550*<br>*(0.0540)*	0.0702<br>*0.1194*<br>*(0.0382)*	0.0775<br>*0.1382*<br>*(0.0602)*
$h_1 = 4$	0.2280<br>*0.3578*<br>*(0.1511)*	1.8481<br>*1.5049*<br>*(0.2793)*	0.1255<br>*0.2027*<br>*(0.0839)*	0.0741<br>*0.1423*<br>*(0.0556)*	0.0768<br>*0.1859*<br>*(0.0754)*
$h_1 = 5$	0.2560<br>*0.3885*<br>*(0.1392)*	1.9155<br>*1.8982*<br>*(0.2292)*	0.3033<br>*0.3764*<br>*(0.1611)*	0.0402<br>*0.1642*<br>*(0.0814)*	0.0962<br>*0.2138*<br>*(0.1064)*
$\lambda^{(2)}_{13,h_1,h_2}$					
$h_1 = 1$	0.6392<br>*0.5293*<br>*(0.1450)*	1.0358<br>*0.9440*<br>*(0.2261)*	0.2166<br>*0.2590*<br>*(0.1078)*	0.2147<br>*0.3597*<br>*(0.1479)*	0.7352<br>*0.8130*<br>*(0.2035)*
$h_1 = 2$	0.0835<br>*0.4594*<br>*(0.2109)*	0.8022<br>*0.3897*<br>*(0.1680)*	0.0578<br>*0.2384*<br>*(0.1032)*	0.5010<br>*0.4358*<br>*(0.1664)*	0.6139<br>*0.4166*<br>*(0.1844)*
$h_1 = 3$	1.1690<br>*1.1939*<br>*(0.2850)*	0.0101<br>*0.2267*<br>*(0.0837)*	0.0046<br>*0.1511*<br>*(0.0513)*	0.2853<br>*0.3055*<br>*(0.1203)*	0.0249<br>*0.2092*<br>*(0.0852)*
$h_1 = 4$	1.4328<br>*1.5554*<br>*(0.2518)*	1.4463<br>*1.3057*<br>*(0.2743)*	0.2949<br>*0.3470*<br>*(0.1421)*	0.3306<br>*0.4160*<br>*(0.1570)*	0.2793<br>*0.2857*<br>*(0.1175)*
$h_1 = 5$	0.2204<br>*0.3097*<br>*(0.1194)*	0.5542<br>*0.4521*<br>*(0.1436)*	0.2129<br>*0.2354*<br>*(0.0929)*	0.2910<br>*0.3310*<br>*(0.1403)*	0.0240<br>*0.2037*<br>*(0.0943)*
$\lambda^{(2)}_{14,h_1,h_2}$					
$h_1 = 1$	0.1655<br>*0.2011*<br>*(0.0753)*	0.3246<br>*0.2310*<br>*(0.1005)*	0.2066<br>*0.2520*<br>*(0.0987)*	0.0748<br>*0.1828*<br>*(0.0776)*	0.5702<br>*0.5528*<br>*(0.2067)*
$h_1 = 2$	0.1766<br>*0.2845*<br>*(0.1049)*	0.3546<br>*0.2848*<br>*(0.1130)*	0.2947<br>*0.3467*<br>*(0.1256)*	0.1471<br>*0.4064*<br>*(0.2114)*	0.6335<br>*0.5351*<br>*(0.2606)*
$h_1 = 3$	0.1988<br>*0.2417*<br>*(0.1023)*	0.3046<br>*0.3200*<br>*(0.1251)*	0.3038<br>*0.5843*<br>*(0.2199)*	1.0917<br>*0.9380*<br>*(0.2789)*	0.1258<br>*0.3597*<br>*(0.1589)*
$h_1 = 4$	0.3164<br>*0.2164*<br>*(0.0859)*	0.0997<br>*0.3213*<br>*(0.1488)*	1.6758<br>*1.9471*<br>*(0.2563)*	0.3601<br>*0.3712*<br>*(0.1656)*	0.0692<br>*0.3569*<br>*(0.1735)*
$h_1 = 5$	1.9246<br>*2.0147*<br>*(0.2185)*	0.0327<br>*0.1505*<br>*(0.0526)*	0.1673<br>*0.2626*<br>*(0.1175)*	0.4517<br>*0.5705*<br>*(0.2252)*	0.9108<br>*0.9485*<br>*(0.2512)*
$\lambda^{(2)}_{15,h_1,h_2}$					
$h_1 = 1$	0.0498<br>*0.1002*<br>*(0.0461)*	0.1985<br>*0.2524*<br>*(0.0959)*	0.9389<br>*1.1316*<br>*(0.1852)*	0.1364<br>*0.1584*<br>*(0.0708)*	0.1251<br>*0.1869*<br>*(0.0831)*
$h_1 = 2$	0.2924<br>*0.2752*<br>*(0.1291)*	0.0388<br>*0.2769*<br>*(0.1138)*	0.3963<br>*0.4583*<br>*(0.1785)*	0.3096<br>*0.2766*<br>*(0.1030)*	0.1663<br>*0.3477*<br>*(0.1285)*
$h_1 = 3$	0.4037<br>*0.3473*<br>*(0.1273)*	0.4108<br>*0.2979*<br>*(0.1067)*	0.1764<br>*0.2842*<br>*(0.1129)*	0.0324<br>*0.1968*<br>*(0.0787)*	0.4337<br>*0.3440*<br>*(0.1353)*
$h_1 = 4$	0.2157<br>*0.1808*<br>*(0.0763)*	0.2640<br>*0.1684*<br>*(0.0723)*	0.0074<br>*0.1402*<br>*(0.0493)*	0.1782<br>*0.1465*<br>*(0.0624)*	0.0339<br>*0.1332*<br>*(0.0498)*
$h_1 = 5$	1.2372<br>*1.3350*<br>*(0.1596)*	0.0350<br>*0.1811*<br>*(0.0763)*	0.3496<br>*0.3773*<br>*(0.1443)*	0.2379<br>*0.2564*<br>*(0.1023)*	0.0863<br>*0.1813*<br>*(0.0723)*
$\lambda^{(2)}_{16,h_1,h_2}$					
$h_1 = 1$	1.9947<br>*1.9083*<br>*(0.2152)*	0.9534<br>*0.8123*<br>*(0.1813)*	0.2755<br>*0.3514*<br>*(0.1461)*	0.0483<br>*0.1813*<br>*(0.0616)*	0.1662<br>*0.1786*<br>*(0.0689)*
$h_1 = 2$	0.1069<br>*0.4922*<br>*(0.2316)*	1.5119<br>*1.0736*<br>*(0.3317)*	0.1957<br>*0.2995*<br>*(0.1084)*	0.0786<br>*0.3093*<br>*(0.1143)*	0.0138<br>*0.4262*<br>*(0.1776)*
$h_1 = 3$	0.5421<br>*0.5644*<br>*(0.2191)*	0.6864<br>*0.4981*<br>*(0.2103)*	0.0017<br>*0.3244*<br>*(0.1270)*	0.4595<br>*0.6064*<br>*(0.2202)*	1.2136<br>*1.1365*<br>*(0.3241)*
$h_1 = 4$	0.2318<br>*0.2778*<br>*(0.1373)*	0.0022<br>*0.2051*<br>*(0.0726)*	0.8042<br>*0.7920*<br>*(0.2274)*	0.8805<br>*0.7498*<br>*(0.2116)*	0.1519<br>*0.3683*<br>*(0.1329)*
$h_1 = 5$	0.1867<br>*0.2554*<br>*(0.1117)*	0.2116<br>*0.3331*<br>*(0.1299)*	0.4981<br>*0.5950*<br>*(0.2055)*	0.6703<br>*0.7099*<br>*(0.2217)*	0.4258<br>*0.6257*<br>*(0.2174)*
$\lambda^{(2)}_{17,h_1,h_2}$					
$h_1 = 1$	1.0531<br>*1.0738*<br>*(0.1602)*	0.5673<br>*0.3963*<br>*(0.1377)*	0.0074<br>*0.1319*<br>*(0.0587)*	0.0329<br>*0.1621*<br>*(0.0628)*	0.3887<br>*0.2965*<br>*(0.1204)*
$h_1 = 2$	0.9862<br>*0.7868*<br>*(0.2264)*	0.4253<br>*0.3855*<br>*(0.1390)*	0.1396<br>*0.3081*<br>*(0.1198)*	0.3706<br>*0.3369*<br>*(0.1349)*	0.3541<br>*0.5710*<br>*(0.2231)*
$h_1 = 3$	0.2542<br>*0.2880*<br>*(0.1193)*	0.1720<br>*0.2858*<br>*(0.0992)*	0.2782<br>*0.2801*<br>*(0.1205)*	0.2978<br>*0.4104*<br>*(0.1747)*	0.6842<br>*0.7238*<br>*(0.2153)*
$h_1 = 4$	0.2270<br>*0.2944*<br>*(0.1198)*	0.6901<br>*0.5376*<br>*(0.1548)*	0.0266<br>*0.1765*<br>*(0.0735)*	0.5626<br>*0.4045*<br>*(0.1481)*	0.3009<br>*0.4218*<br>*(0.1656)*
$h_1 = 5$	0.0691<br>*0.1471*<br>*(0.0594)*	0.2059<br>*0.3013*<br>*(0.1054)*	0.6287<br>*0.6792*<br>*(0.1795)*	0.0267<br>*0.1762*<br>*(0.0670)*	0.2534<br>*0.3874*<br>*(0.1726)*
$\lambda^{(2)}_{18,h_1,h_2}$					
$h_1 = 1$	0.5597<br>*0.4802*<br>*(0.1238)*	0.1824<br>*0.2829*<br>*(0.1165)*	0.7381<br>*0.8420*<br>*(0.1993)*	0.1669<br>*0.1583*<br>*(0.0561)*	0.5369<br>*0.5182*<br>*(0.1811)*
$h_1 = 2$	0.5668<br>*0.4435*<br>*(0.1826)*	0.4077<br>*0.3968*<br>*(0.1709)*	0.0120<br>*0.2855*<br>*(0.1082)*	0.0028<br>*0.1912*<br>*(0.0705)*	0.2128<br>*0.5490*<br>*(0.2376)*
$h_1 = 3$	0.0879<br>*0.2001*<br>*(0.0896)*	0.8780<br>*0.6589*<br>*(0.2012)*	0.1606<br>*0.4320*<br>*(0.2400)*	0.0566<br>*0.1993*<br>*(0.1003)*	1.1111<br>*1.0767*<br>*(0.3193)*
$h_1 = 4$	0.0797<br>*0.0901*<br>*(0.0287)*	0.2492<br>*0.3213*<br>*(0.1348)*	0.9428<br>*1.0673*<br>*(0.2544)*	0.1344<br>*0.1449*<br>*(0.0582)*	0.0973<br>*0.3045*<br>*(0.1372)*
$h_1 = 5$	0.0729<br>*0.1265*<br>*(0.0616)*	0.4228<br>*0.4610*<br>*(0.1589)*	0.4359<br>*0.5631*<br>*(0.1837)*	0.0062<br>*0.1166*<br>*(0.0511)*	0.4034<br>*0.4743*<br>*(0.1681)*
$\lambda^{(2)}_{19,h_1,h_2}$					
$h_1 = 1$	0.1745<br>*0.2051*<br>*(0.1038)*	0.1248<br>*0.3100*<br>*(0.1164)*	1.2250<br>*1.3788*<br>*(0.2329)*	0.6456<br>*0.5670*<br>*(0.1954)*	0.4731<br>*0.6483*<br>*(0.2329)*
$h_1 = 2$	2.1188<br>*1.6541*<br>*(0.2825)*	0.7801<br>*0.6591*<br>*(0.2493)*	0.1350<br>*0.3389*<br>*(0.1308)*	0.1734<br>*0.5341*<br>*(0.2130)*	0.3241<br>*0.4629*<br>*(0.1916)*
$h_1 = 3$	0.0508<br>*0.2287*<br>*(0.1788)*	0.7314<br>*0.4667*<br>*(0.1895)*	0.0803<br>*0.2454*<br>*(0.0917)*	0.9953<br>*0.9324*<br>*(0.2453)*	0.1206<br>*0.3537*<br>*(0.1516)*
$h_1 = 4$	0.0577<br>*0.1206*<br>*(0.0412)*	0.3197<br>*0.2524*<br>*(0.1020)*	0.2578<br>*0.2885*<br>*(0.1138)*	0.6508<br>*0.6470*<br>*(0.2155)*	0.3765<br>*0.4400*<br>*(0.1893)*
$h_1 = 5$	0.0490<br>*0.1201*<br>*(0.0498)*	0.0184<br>*0.1389*<br>*(0.0511)*	0.1220<br>*0.1577*<br>*(0.0642)*	0.0905<br>*0.2141*<br>*(0.1021)*	0.4323<br>*0.3773*<br>*(0.1437)*
$\lambda^{(2)}_{20,h_1,h_2}$					
$h_1 = 1$	0.0930<br>*0.0971*<br>*(0.0347)*	0.1313<br>*0.1175*<br>*(0.0541)*	0.2267<br>*0.1981*<br>*(0.0887)*	0.0413<br>*0.1100*<br>*(0.0396)*	0.6160<br>*0.4814*<br>*(0.1442)*
$h_1 = 2$	0.5845<br>*0.6095*<br>*(0.3009)*	0.2108<br>*0.2379*<br>*(0.0897)*	0.0591<br>*0.3306*<br>*(0.1388)*	0.0164<br>*0.2666*<br>*(0.1281)*	0.0794<br>*0.2896*<br>*(0.1229)*
$h_1 = 3$	1.4735<br>*1.6014*<br>*(0.2927)*	0.2103<br>*0.3109*<br>*(0.1303)*	0.5768<br>*0.6276*<br>*(0.2316)*	0.9584<br>*1.0073*<br>*(0.3132)*	0.1616<br>*0.3640*<br>*(0.1608)*
$h_1 = 4$	0.4664<br>*0.4090*<br>*(0.1462)*	0.2640<br>*0.2375*<br>*(0.0944)*	0.1660<br>*0.2828*<br>*(0.1211)*	0.7746<br>*0.6703*<br>*(0.2107)*	0.4399<br>*0.4773*<br>*(0.1697)*
$h_1 = 5$	0.2224<br>*0.3597*<br>*(0.1381)*	0.5343<br>*0.5295*<br>*(0.1383)*	0.3554<br>*0.4097*<br>*(0.1528)*	0.0796<br>*0.2040*<br>*(0.0833)*	0.0349<br>*0.1670*<br>*(0.0579)*

Table 3. Comparison between true $\lambda^{(3)}_{k,h_2}, k = 1, \ldots, K, h_2 = 1, \ldots, H_2$ and the estimated values from the BPRTTD model in terms of posterior mean. Average (*italic*) and standard deviation (*italic* in parentheses) of the posterior means over 100 repetitions are reported.

$\lambda^{(3)}_{k,h_2}$	$h_2 = 1$	$h_2 = 2$	$h_2 = 3$	$h_2 = 4$	$h_2 = 5$		$h_2 = 1$	$h_2 = 2$	$h_2 = 3$	$h_2 = 4$	$h_2 = 5$
	1.4632	0.4675	0.0503	0.3455	0.3300		0.6673	0.2659	0.2560	0.1324	0.0326
$k = 1$	*1.3773*	*0.3533*	*0.0808*	*0.1922*	*0.2828*	$k = 11$	*0.6076*	*0.2287*	*0.1629*	*0.0967*	*0.0611*
	(*0.1148*)	(*0.1003*)	(*0.0336*)	(*0.0698*)	(*0.0757*)		(*0.0628*)	(*0.0547*)	(*0.0541*)	(*0.0372*)	(*0.0290*)
	1.5325	0.3920	0.1011	0.3003	0.3005		0.1721	0.1113	0.1160	0.7142	0.2496
$k = 2$	*1.4338*	*0.2843*	*0.0949*	*0.1699*	*0.2640*	$k = 12$	*0.1504*	*0.0746*	*0.0895*	*0.6215*	*0.1902*
	(*0.1326*)	(*0.0812*)	(*0.0382*)	(*0.0686*)	(*0.0769*)		(*0.0477*)	(*0.0346*)	(*0.0408*)	(*0.0639*)	(*0.0586*)
	0.7693	0.1223	0.1671	0.2508	0.0306		0.0813	0.1478	1.0926	0.8203	0.6685
$k = 3$	*0.6930*	*0.0863*	*0.1207*	*0.1735*	*0.0664*	$k = 13$	*0.0759*	*0.0935*	*0.7440*	*0.7640*	*0.5372*
	(*0.0651*)	(*0.0349*)	(*0.0473*)	(*0.0521*)	(*0.0299*)		(*0.0284*)	(*0.0310*)	(*0.1069*)	(*0.1024*)	(*0.0886*)
	0.3379	0.7165	0.6490	0.2529	0.2264		0.0407	0.3713	0.5543	0.4727	0.1632
$k = 4$	*0.3163*	*0.7006*	*0.3853*	*0.2294*	*0.2186*	$k = 14$	*0.0554*	*0.3453*	*0.3516*	*0.4412*	*0.1299*
	(*0.0649*)	(*0.0853*)	(*0.0797*)	(*0.0597*)	(*0.0667*)		(*0.0264*)	(*0.0560*)	(*0.0655*)	(*0.0582*)	(*0.0499*)
	0.3874	0.7133	0.6555	0.0439	0.2120		0.1495	1.3263	1.0017	0.2329	0.1638
$k = 5$	*0.3626*	*0.6852*	*0.3902*	*0.0811*	*0.1921*	$k = 15$	*0.1499*	*1.3300*	*0.5778*	*0.2522*	*0.1727*
	(*0.0682*)	(*0.0808*)	(*0.0764*)	(*0.0379*)	(*0.0528*)		(*0.0548*)	(*0.1294*)	(*0.1164*)	(*0.0973*)	(*0.0688*)
	0.0672	0.3671	0.4781	0.2684	1.3715		0.1958	0.4301	1.1088	0.0860	0.3803
$k = 6$	*0.1205*	*0.2926*	*0.2779*	*0.2015*	*1.1118*	$k = 16$	*0.1770*	*0.4067*	*0.7255*	*0.1239*	*0.3397*
	(*0.0506*)	(*0.0782*)	(*0.0859*)	(*0.0750*)	(*0.1054*)		(*0.0583*)	(*0.0782*)	(*0.0912*)	(*0.0491*)	(*0.0751*)
	0.4289	0.9567	0.8054	0.3852	0.0244		0.2621	0.1393	0.0465	0.7282	0.4688
$k = 7$	*0.3738*	*0.9383*	*0.4935*	*0.3591*	*0.0772*	$k = 17$	*0.2425*	*0.0925*	*0.0628*	*0.5906*	*0.3632*
	(*0.0847*)	(*0.1042*)	(*0.0929*)	(*0.0815*)	(*0.0364*)		(*0.0597*)	(*0.0404*)	(*0.0258*)	(*0.0834*)	(*0.0829*)
	0.2670	0.7717	0.5174	0.3743	0.3509		0.1474	0.1712	1.5260	0.3272	0.0382
$k = 8$	*0.2562*	*0.7508*	*0.2997*	*0.3271*	*0.3046*	$k = 18$	*0.0941*	*0.1626*	*1.0389*	*0.3609*	*0.0843*
	(*0.0665*)	(*0.0825*)	(*0.0778*)	(*0.0672*)	(*0.0691*)		(*0.0390*)	(*0.0585*)	(*0.1046*)	(*0.0910*)	(*0.0342*)
	0.0633	0.4225	0.6116	0.0402	0.6689		0.1874	0.5248	0.1737	0.6770	0.0303
$k = 9$	*0.0889*	*0.3743*	*0.3555*	*0.0742*	*0.5528*	$k = 19$	*0.1676*	*0.4967*	*0.0853*	*0.5875*	*0.0588*
	(*0.0369*)	(*0.0659*)	(*0.0728*)	(*0.0310*)	(*0.0703*)		(*0.0595*)	(*0.0678*)	(*0.0350*)	(*0.0696*)	(*0.0272*)
	0.0545	0.0128	0.2266	0.2864	0.3887		0.6216	0.1774	0.0174	0.5919	0.2238
$k = 10$	*0.0432*	*0.0191*	*0.1429*	*0.2407*	*0.3111*	$k = 20$	*0.5772*	*0.1168*	*0.0565*	*0.4612*	*0.1698*
	(*0.0180*)	(*0.0063*)	(*0.0407*)	(*0.0480*)	(*0.0530*)		(*0.0700*)	(*0.0489*)	(*0.0267*)	(*0.0669*)	(*0.0613*)

Table 4. Summary statistics of deviance statistic over 100 repetitions.

	Min.	1st Qu.	Median	Mean	3rd Qu.	Max.
GLM	51419	211978	213434	212776	214576	218440
BPRCPD	9351	96686	97669	96903	99024	101516
BPRTTD	7962	95426	96349	95596	97618	100352

2. Poisson regression coefficients

The following table displays the posterior mean estimates of the Poisson regression coefficients in the BPRTTD model as well as the 95% credible intervals.

Table 5. Posterior mean estimates $\hat{\boldsymbol{\beta}}$ of the BPRTTD model with 95% credible intervals.

Coefficients	Posterior mean	95% CI lower bound	95% CI upper bound
Intercept	-16.6495	-16.6527	-16.6462
isi	0.0622	0.0622	0.0623
causes2	-0.6237	-0.6263	-0.6212
causes3	1.8678	1.8639	1.8717
causes4	-2.0146	-2.0214	-2.0086
causes5	-0.5788	-0.5821	-0.5758
causes6	-1.4547	-1.4584	-1.4512
causes7	-0.2957	-0.2997	-0.2917
causes8	1.0847	1.0827	1.0866
causes9	-0.6687	-0.6706	-0.6667
causes10	-0.2185	-0.2217	-0.2157
causes11	-4.3441	-4.3581	-4.3305
causes12	-2.3376	-2.3408	-2.3349
causes13	-2.4258	-2.4266	-2.4250
causes14	-4.3635	-4.3723	-4.3543
causes15	-0.2488	-0.2517	-0.2457
causes16	-0.5248	-0.5302	-0.5194
causes17	-0.0979	-0.0999	-0.0958
causes18	-3.1404	-3.1431	-3.1377
age50-59	1.1601	1.1568	1.1628
age60-64	1.2391	1.2361	1.2417
age65-69	1.6878	1.6830	1.6921
age70-74	2.2840	2.2790	2.2883
age75-79	2.6251	2.6202	2.6292
age80-84	3.0926	3.0875	3.0970
age85-89	3.1920	3.1873	3.1962
age90-94	2.9043	2.9002	2.9077
age95+	2.0729	2.0680	2.0772
sexmale	0.5431	0.5423	0.5437
isi:causes2	-0.0642	-0.0642	-0.0642
isi:causes3	-0.0634	-0.0634	-0.0634
isi:causes4	-0.0618	-0.0619	-0.0618
isi:causes5	-0.0607	-0.0607	-0.0607
isi:causes6	-0.0614	-0.0614	-0.0613
isi:causes7	-0.0613	-0.0613	-0.0612
isi:causes8	-0.0634	-0.0635	-0.0634
isi:causes9	-0.0611	-0.0611	-0.0610
isi:causes10	-0.0636	-0.0636	-0.0636
isi:causes11	-0.0622	-0.0623	-0.0622
isi:causes12	-0.0613	-0.0613	-0.0613
isi:causes13	-0.0613	-0.0613	-0.0612
isi:causes14	-0.0676	-0.0678	-0.0673
isi:causes15	-0.0648	-0.0648	-0.0648
isi:causes16	-0.0628	-0.0629	-0.0628
isi:causes17	-0.0547	-0.0547	-0.0547
isi:causes18	-0.0598	-0.0598	-0.0598
causes2:age50-59	0.5114	0.5091	0.5141
causes3:age50-59	0.9742	0.9713	0.9776
causes4:age50-59	0.0313	0.0236	0.0400
causes5:age50-59	0.7903	0.7860	0.7953
causes6:age50-59	0.3711	0.3687	0.3741
causes7:age50-59	0.3307	0.3275	0.3344

Coefficients	Posterior mean	95% CI lower bound	95% CI upper bound
causes8:age50-59	0.8084	0.8063	0.8110
causes9:age50-59	0.6622	0.6597	0.6653
causes10:age50-59	0.8108	0.8066	0.8155
causes11:age50-59	0.9046	0.8903	0.9187
causes12:age50-59	0.5281	0.5240	0.5331
causes13:age50-59	0.9430	0.9406	0.9456
causes14:age50-59	-3.1612	-3.1970	-3.1241
causes15:age50-59	-4.1632	-4.1697	-4.1555
causes16:age50-59	-0.8207	-0.8271	-0.8139
causes17:age50-59	-0.2131	-0.2152	-0.2105
causes18:age50-59	-0.6158	-0.6190	-0.6121
causes2:age60-64	0.7662	0.7627	0.7700
causes3:age60-64	1.5958	1.5922	1.5999
causes4:age60-64	0.4112	0.4077	0.4152
causes5:age60-64	1.4999	1.4961	1.5044
causes6:age60-64	0.8541	0.8488	0.8599
causes7:age60-64	0.9691	0.9661	0.9725
causes8:age60-64	1.4578	1.4562	1.4597
causes9:age60-64	1.5201	1.5192	1.5213
causes10:age60-64	1.2630	1.2594	1.2670
causes11:age60-64	1.6019	1.5821	1.6238
causes12:age60-64	1.1331	1.1290	1.1377
causes13:age60-64	1.7587	1.7559	1.7619
causes14:age60-64	-3.9336	-5.1843	-2.8401
causes15:age60-64	-4.5475	-4.5634	-4.5321
causes16:age60-64	-0.5220	-0.5274	-0.5170
causes17:age60-64	-0.0207	-0.0212	-0.0202
causes18:age60-64	-0.5913	-0.5937	-0.5886
causes2:age65-69	0.7445	0.7424	0.7470
causes3:age65-69	1.5623	1.5575	1.5677
causes4:age65-69	0.4822	0.4714	0.4948
causes5:age65-69	1.5700	1.5653	1.5754
causes6:age65-69	0.8514	0.8467	0.8570
causes7:age65-69	1.1071	1.1011	1.1139
causes8:age65-69	1.5204	1.5172	1.5241
causes9:age65-69	1.7369	1.7336	1.7406
causes10:age65-69	1.1631	1.1590	1.1679
causes11:age65-69	1.7110	1.7003	1.7220
causes12:age65-69	1.2160	1.2120	1.2207
causes13:age65-69	1.9535	1.9495	1.9580
causes14:age65-69	-2.9935	-4.1278	-2.0717
causes15:age65-69	-5.0895	-5.1082	-5.0735
causes16:age65-69	-1.3104	-1.3187	-1.3012
causes17:age65-69	-0.2727	-0.2760	-0.2689
causes18:age65-69	-0.9068	-0.9110	-0.9019
causes2:age70-74	0.7922	0.7892	0.7956
causes3:age70-74	1.4096	1.4046	1.4152
causes4:age70-74	0.4786	0.4725	0.4857
causes5:age70-74	1.5377	1.5339	1.5421
causes6:age70-74	1.1036	1.0975	1.1105
causes7:age70-74	1.2139	1.2093	1.2192
causes8:age70-74	1.5491	1.5456	1.5532

Coefficients	Posterior mean	95% CI lower bound	95% CI upper bound
causes9:age70-74	1.9028	1.9003	1.9054
causes10:age70-74	1.0739	1.0697	1.0788
causes11:age70-74	1.8440	1.8273	1.8612
causes12:age70-74	1.1411	1.1357	1.1473
causes13:age70-74	2.0869	2.0844	2.0897
causes14:age70-74	-5.2264	-6.4161	-4.2635
causes15:age70-74	-6.3054	-6.3257	-6.2844
causes16:age70-74	-2.0003	-2.0094	-1.9908
causes17:age70-74	-0.4288	-0.4344	-0.4225
causes18:age70-74	-1.0744	-1.0783	-1.0699
causes2:age75-79	1.1387	1.1349	1.1432
causes3:age75-79	1.4602	1.4556	1.4656
causes4:age75-79	0.8348	0.8273	0.8435
causes5:age75-79	1.8020	1.7975	1.8074
causes6:age75-79	1.8753	1.8706	1.8808
causes7:age75-79	1.6761	1.6709	1.6823
causes8:age75-79	1.9453	1.9419	1.9492
causes9:age75-79	2.3313	2.3286	2.3347
causes10:age75-79	1.2609	1.2573	1.2653
causes11:age75-79	2.2776	2.2641	2.2915
causes12:age75-79	1.4373	1.4326	1.4431
causes13:age75-79	2.5700	2.5670	2.5734
causes14:age75-79	-2.6592	-3.4425	-2.0250
causes15:age75-79	-6.8405	-6.8979	-6.7823
causes16:age75-79	-2.3272	-2.3337	-2.3197
causes17:age75-79	-0.2369	-0.2406	-0.2326
causes18:age75-79	-0.8145	-0.8179	-0.8105
causes2:age80-84	1.4811	1.4771	1.4859
causes3:age80-84	1.4177	1.4130	1.4231
causes4:age80-84	1.1926	1.1850	1.2016
causes5:age80-84	2.0797	2.0753	2.0849
causes6:age80-84	2.6113	2.6070	2.6165
causes7:age80-84	1.9817	1.9770	1.9872
causes8:age80-84	2.3790	2.3758	2.3828
causes9:age80-84	2.7303	2.7271	2.7341
causes10:age80-84	1.4668	1.4625	1.4719
causes11:age80-84	2.7364	2.7226	2.7509
causes12:age80-84	1.7016	1.6972	1.7068
causes13:age80-84	3.0390	3.0357	3.0426
causes14:age80-84	-7.7412	-8.3177	-7.1522
causes15:age80-84	-6.4995	-6.5073	-6.4923
causes16:age80-84	-2.4258	-2.4336	-2.4168
causes17:age80-84	0.2797	0.2759	0.2843
causes18:age80-84	-0.4742	-0.4781	-0.4696
causes2:age85-89	2.0728	2.0693	2.0770
causes3:age85-89	1.6626	1.6577	1.6679
causes4:age85-89	1.9703	1.9619	1.9802
causes5:age85-89	2.6874	2.6836	2.6918
causes6:age85-89	3.5980	3.5931	3.6036
causes7:age85-89	2.5000	2.4955	2.5050
causes8:age85-89	3.1934	3.1904	3.1970
causes9:age85-89	3.4762	3.4733	3.4795

Coefficients	Posterior mean	95% CI lower bound	95% CI upper bound
causes10:age85-89	1.9912	1.9871	1.9961
causes11:age85-89	3.5340	3.5208	3.5482
causes12:age85-89	2.2547	2.2510	2.2590
causes13:age85-89	3.8594	3.8573	3.8618
causes14:age85-89	-3.1789	-4.1169	-2.3403
causes15:age85-89	-6.4308	-6.4654	-6.3910
causes16:age85-89	-2.1175	-2.1227	-2.1124
causes17:age85-89	1.3042	1.3003	1.3087
causes18:age85-89	0.2321	0.2288	0.2358
causes2:age90-94	2.8457	2.8421	2.8501
causes3:age90-94	2.1375	2.1334	2.1422
causes4:age90-94	3.0483	3.0421	3.0555
causes5:age90-94	3.6021	3.5989	3.6061
causes6:age90-94	4.7759	4.7716	4.7809
causes7:age90-94	3.1840	3.1801	3.1885
causes8:age90-94	4.2905	4.2882	4.2933
causes9:age90-94	4.4775	4.4752	4.4803
causes10:age90-94	2.8103	2.8061	2.8152
causes11:age90-94	4.6064	4.5920	4.6218
causes12:age90-94	3.2598	3.2566	3.2637
causes13:age90-94	4.8899	4.8881	4.8919
causes14:age90-94	-11.6281	-12.6193	-10.6308
causes15:age90-94	-7.1391	-8.4802	-5.9674
causes16:age90-94	-1.4710	-1.4774	-1.4636
causes17:age90-94	2.8321	2.8292	2.8355
causes18:age90-94	1.2644	1.2612	1.2682
causes2:age95+	4.0183	4.0125	4.0251
causes3:age95+	3.0521	3.0477	3.0572
causes4:age95+	4.5687	4.5607	4.5781
causes5:age95+	4.9195	4.9142	4.9259
causes6:age95+	6.3040	6.2993	6.3093
causes7:age95+	4.2865	4.2815	4.2923
causes8:age95+	5.8169	5.8139	5.8205
causes9:age95+	5.9212	5.9184	5.9248
causes10:age95+	4.0414	4.0375	4.0459
causes11:age95+	6.2110	6.1931	6.2306
causes12:age95+	4.7972	4.7923	4.8030
causes13:age95+	6.2951	6.2933	6.2972
causes14:age95+	-1.3673	-2.8763	-0.0799
causes15:age95+	-4.7655	-4.7879	-4.7441
causes16:age95+	-0.3515	-0.3545	-0.3483
causes17:age95+	4.9397	4.9366	4.9433
causes18:age95+	2.6959	2.6925	2.6996
age50-59:sexmale	0.0042	0.0036	0.0049
age60-64:sexmale	0.0656	0.0653	0.0660
age65-69:sexmale	0.0853	0.0850	0.0857
age70-74:sexmale	0.0579	0.0575	0.0584
age75-79:sexmale	-0.0080	-0.0085	-0.0074
age80-84:sexmale	-0.1175	-0.1184	-0.1164
age85-89:sexmale	-0.2113	-0.2119	-0.2106
age90-94:sexmale	-0.3004	-0.3014	-0.2994
age95+:sexmale	-0.4041	-0.4049	-0.4031
